# Green phytofabrication of silver nanoparticles from underexplored cypriot plant species with tunable antimicrobial, antioxidant and anticancer activities

**DOI:** 10.1039/d6ra06253a

**Published:** 2026-10-21

**Authors:** Doga Kavaz, Temitayo Margaret Omoyeni, Buse Taskiranlar, Huzaifa Umar

**Affiliations:** a Department of Bioengineering, Faculty of Engineering, Cyprus International University North Cyprus, Via Mersin 10 Haspolat Nicosia Turkey tomoyeni@ciu.edu.tr; b Biotechnology Research Centre, Cyprus International University North Cyprus, Via Mersin 10 Haspolat Nicosia Turkey; c Department of Basic Sciences and Humanities, Faculty of Arts and Sciences, Cyprus International University North Cyprus, Via Mersin 10 Haspolat Nicosia Turkey; d Irfan Suat Gunsel, Operational Research Institute, Near East University TRNC Mersin 10 Nicosia 99138 Turkey; e Jadara Research Center, Jadara University Jordan

## Abstract

The chemical stability and biocompatibility of nanoparticles produced from plant extracts indicate medicinal implications, rendering them suitable candidates for antibacterial and antioxidant applications. This study aimed to synthesize AgNPs using *A. andrachne* and *P. lentiscus* leaf extracts and compare their antioxidant, antibacterial, and anticancer activities. This study presented the green synthesis of highly stable silver nanoparticles, measuring around 90.87 nm and 78.26 nm, derived from the extracts of *Arbutus andrachne* and *Pistacia lentiscus*, respectively. The surface compositional analysis of the synthesized silver nanoparticles (AgNPs) was conducted using UV-visible spectroscopy, Fourier transform infrared spectroscopy, X-ray diffraction, zeta potential analysis, energy-dispersive X-ray spectroscopy, and scanning electron microscopy. Gas chromatography-mass spectrometry analysis was conducted to separate and detect the chemical components of the plant extracts. The antimicrobial efficacy of AgNPs was demonstrated against *Escherichia coli* and *Enterococcus faecalis*. Both samples showed significant cytotoxicity against a cancer cell line, with cell viability decreasing as AgNP concentration increased, and it was observed that *A. andrachne* AgNPs exhibited stronger cytotoxic effects than *P. lentiscus* AgNPs. Overall, the findings demonstrate the potential of *A. andrachne* and *P. lentiscus* extracts for the green synthesis of biologically active AgNPs.

## Abstract 2

Silver nanoparticles were green-synthesized using *Arbutus andrachne* and *Pistacia lentiscus* leaf extracts and evaluated for antimicrobial, antioxidant, and anticancer activities. The study explores underexamined Cypriot plants as sources of bioactive nanomaterials.

## Graphical abstract

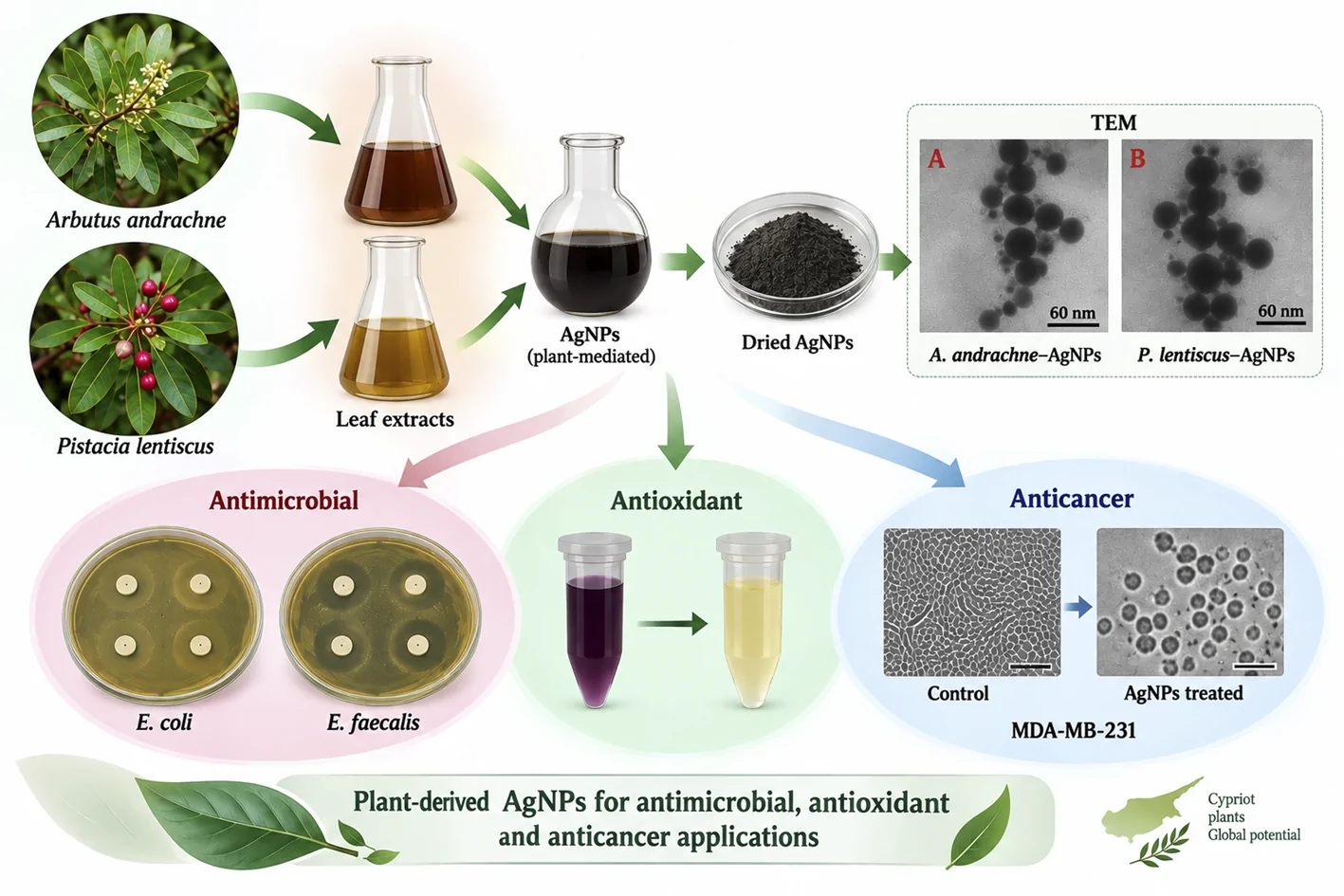

## 1. Introduction

Nanotechnology encompasses diverse domains, including environmental science, agriculture, medicine, and food, and represents major rapidly advancing research sectors amidst the agri-food industry. Advancements in nanotechnology have led to significant growth in the agri-food sector, enhancing global food production in terms of quality, safety, and nutritional value. Recently, the demand for nanomaterials has surged considerably due to the rapidly expanding nanotechnology business.^1^

The green synthesis of silver nanoparticles (AgNPs) is highly beneficial compared to other techniques that utilize microorganisms, especially because it is non-toxic, can easily be enhanced and bypasses the complex stage of growing cell cultures. Most importantly, the process can be seamlessly scaled up for commercial-scale synthesis of NPs.^2^

Silver nanoparticles (AgNPs) are widely known for their catalytic activity, high conductivity, stability, and distinctive optical and electronic characteristics. The physicochemical characteristics of AgNPs, including morphology, surface area, surface charge, dissolution rate, and surface coating, are crucial to biological effects. The utilization of biological reducing agents, for example, culture supernatants from several *Bacillus* species, facilitates the synthesis of AgNPs in diverse morphologies, including rod-like, octagonal, spherical, hexagonal, flower-like, and triangular forms.^3^

*Arbutus andrachne* is a plant that also bears fruits, and it belongs to the Ericaceae family. It is widely found in the Eastern Mediterranean region, mainly in Greece and Turkey, and it is popularly known in the Turkish region as “sandal ağacı”. The fruits of the plant are widely used in making marmalade, fruit jelly and jam in the food industry and in making wine and liquor in the alcoholic beverage industry. The entire plant is used for various ethnomedicinal purposes in the Mediterranean region. The antioxidant activities of the *A. andrachne* methanol extracts from roots, leaves and fruits have been previously investigated, and it has been reported that the methanol extract of the roots exhibits high antioxidant activity and antihypertensive effect.^4^

*Pistacia lentiscus* is a famous plant that is mostly found in the Mediterranean region. It has a promising health-positive profile for boosting respiratory and gastrointestinal health and treating other inflammatory diseases. Recent research shows that essential oils, such as PO, struggle with poor solubility, bioavailability and high volatility, which limit their use in medicine. To overcome such limitations associated with the clinical application of natural extracts and enhance their therapeutic efficacy, considerable attention has been directed toward nanotechnology-based approaches, particularly the plant-mediated synthesis of silver nanoparticles (AgNPs). However, a critical gap remains.^5^

Although the plant-mediated synthesis of silver nanoparticles (AgNPs) has been widely investigated, limited attention has been given to comparing nanoparticles produced from *Arbutus andrachne* and *Pistacia lentiscus* leaf extracts within the same experimental framework. To the best of our knowledge, no previous study has directly compared the antibacterial, antioxidant, and anticancer activities of AgNPs prepared from these two plant species. The significance of this work, therefore, extends beyond the synthesis of AgNPs and focuses on evaluating how the plant source affects their biological performance. By examining the nanoparticles under comparable experimental conditions, the study highlights possible differences associated with the distinct phytochemical composition of each plant extract. The observed variations in biological activity suggest that the selection of plant material can play an important role in determining the functional properties of green-synthesized AgNPs. These findings provide additional insight into plant-dependent differences in AgNP bioactivity and support further investigation of *A. andrachne* and *P. lentiscus* as natural resources for developing multifunctional nanoparticles with potential biomedical applications. *Arbutus andrachne* and *Pistacia lentiscus* leaves are used in this study because of their benign nature and the presence of some essential functional groups that allow AgNPs to be very stable and hinder the agglomeration of binding nanoparticles. This study aims to synthesize AgNPs, which possess eco-friendly and antibacterial properties, using *Arbutus andrachne* and *Pistacia lentiscus* leaf extracts. To investigate the antioxidant activities of the synthesized AgNPs from *Arbutus andrachne* and *Pistacia lentiscus* plants, the free radical scavenging, total antioxidant capacity and metal chelating activity of the samples are evaluated. The characterization of silver nanoparticles is performed by SEM, EDX, TEM, XRD, and FTIR spectroscopy. The optical absorption properties of the sample are measured using a UV-visible spectrophotometer. Additionally, its antibacterial activity is investigated using the disk diffusion method for Gram-positive (*Enterococcus faecalis*) and Gram-negative (*Escherichia coli*) bacteria. Finally, its anticancer potential in the MDA-MB-231 cell line model is evaluated using trypan blue assays. The method in this research is easy, eco-friendly, and non-toxic; requires only non-hazardous reactants, such as plant extract, water, and silver nitrate; and is advantageous in the large-scale production of silver nanoparticles. Hopefully, this will fill in the critical void in the literature and serve as a foundation for further investigations in the field of cancer therapy, antibacterial and antioxidant studies.

## 2. Materials and methods

### 2.1 Materials and chemicals

All reagent-grade substances were directly used in this experiment without any further purification processes. Nutrient agar (MHA), nutrient broth, DPPH, ascorbic acid, antibiotics (azithromycin), swabs, microfilters, and Petri dishes were obtained from Sigma-Aldrich, USA. Silver nitrate, ethanol, DMSO, and methanol were procured from Merck, Germany. Distilled water was procured from the CIU and utilized throughout the investigation.

### 2.2 Plant collection and identification

*Arbutus andrachne* and *Pistacia lentiscus* specimens, characterized by lush green foliage and a pronounced fragrance, were gathered in separate bags from the elevated regions of Alevkayası (35°17′14.6″ N 33°31′48.1″ E) in northern Cyprus. The common name of *Arbutus andrachne* is Greek strawberry tree, and the common name of *Pistacia lentiscus* is mastic tree. *Arbutus andrachne* is from the Ericaceae family, and *Pistacia lentiscus* is from the Anacardiaceae family. The plant identification was done by Associate Prof. Dr Emmanuel Halilu at Cyprus International University (CIU). The voucher specimen was deposited in the herbarium of the Faculty of Pharmacy in Cyprus International University for future reference, with voucher numbers CIU|PHARM|ERIC|001 (*Arbutus andrachne*) and CIU|PHARM|ANAC|001 (*Pistacia lentiscus*).

### 2.3 Plant preparation and extraction

The gathered *Arbutus andrachne* and *Pistacia lentiscus* plants were subjected to sunlight for 2 h daily for a period of 8 days and subsequently desiccated. At the conclusion of the eighth day, the desiccated plants were detached from their leaves and sectioned into relatively small fragments using scissors. The dried plant fragments were pulverized using a blender and stored in two distinct jars based on type.

#### 2.3.1 Extraction process

Initially, 1 : 10 mL of ethanol and distilled water was measured and transferred into an Erlenmeyer flask, then 20 grams of *Pistacia lentiscus* powder was introduced into the flask, and the solution was shaken rigorously to avoid clusters. The flask was then sealed with aluminum foil, as illustrated in Fig. 1. The aforementioned processes, implemented individually for ethanol and methanol, were likewise applied to *Arbutus andrachne* plant material. The prepared flasks were kept at room temperature for 24 h.

**Fig. 1:**
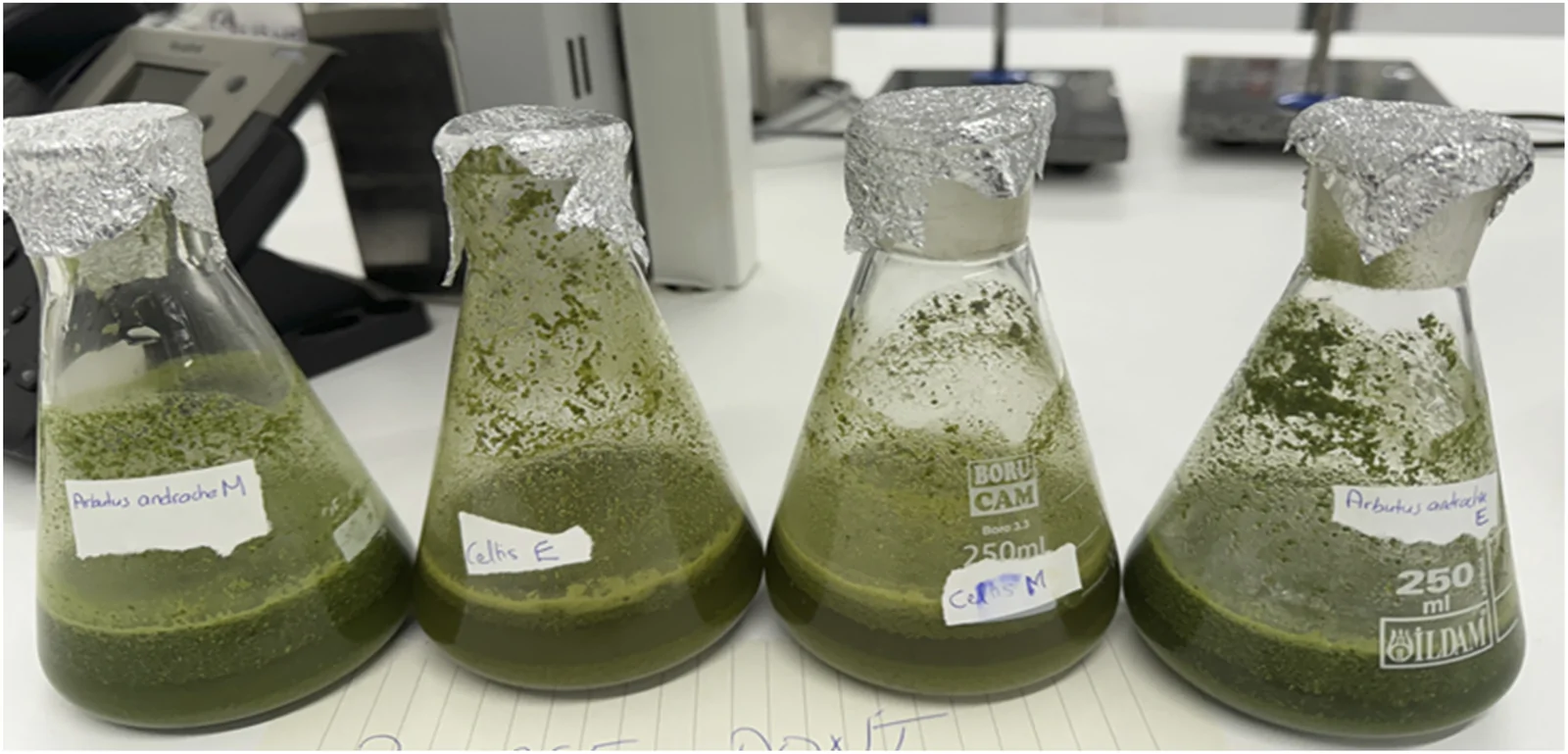
Plant extraction process.

The flask containing all the plant materials was agitated and gradually decanted through filter paper for filtration. The procedure was performed 4–5 times by substituting the filter paper. To achieve the smoothest liquid and to obtain all the components in the plant extract, the process was repeated 5 times, as shown in Fig. 2. The extracted solution was then transferred into a crucible and was placed in an air-drying oven at 30 °C for 48 h. Afterward, the dried extracts were removed from the crucible and placed in a centrifuge tube for further analysis.

**Fig. 2:**
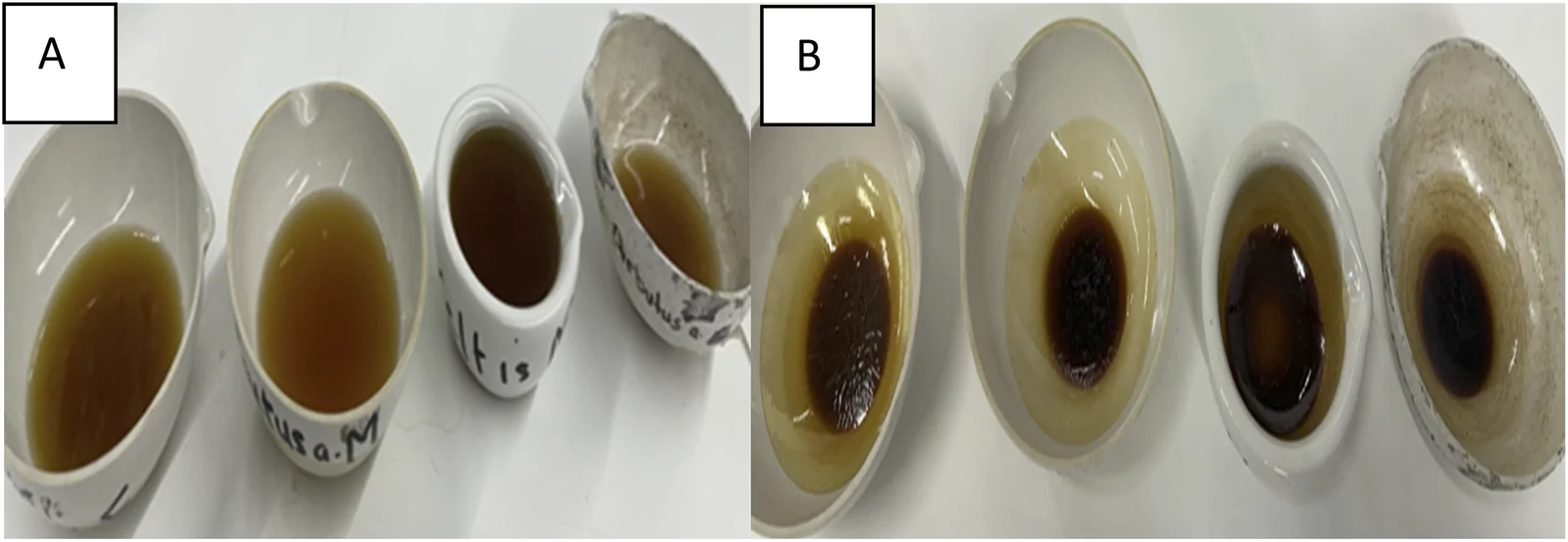
(A) Plant extract before drying and (B) plant extract after drying.

The drying procedure was carried out in an identical sequence for the flasks containing *Arbutus andrachne* E, *Arbutus andrachne* M, *Pistacia lentiscus* E, and *Pistacia lentiscus* M.

### 2.4 Biosynthesis of silver nanoparticles

As shown in Fig. 3, 200 mL of distilled water was transferred into an empty flask, and 20 grams of *Pistacia lentiscus* powder was then added into the flask. The prepared mixture was positioned on a magnetic stirring mantle and subjected to 300 rpm continuous stirring for 24 h. An identical procedure was implemented with *Arbutus andrachne* powder. The *Pistacia lentiscus* mixture was then filtered into another empty flask. The procedure was conducted up to 3 times to achieve a purified liquid, and the flask containing the filtered *Pistacia lentiscus* mixture was sealed with parafilm.

**Fig. 3:**
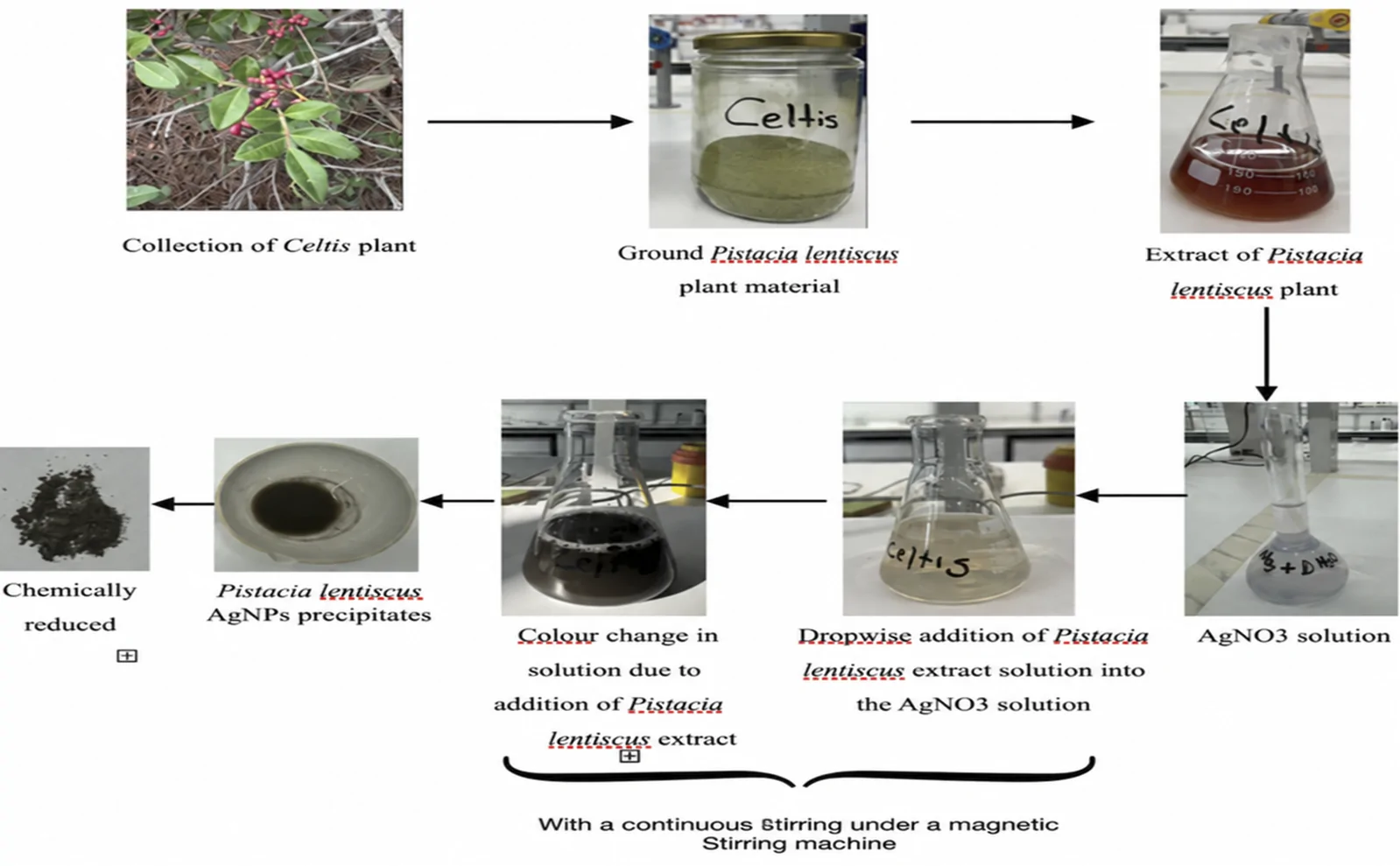
*Pistacia lentiscus* silver nanoparticle synthesis.

A 1.0 mM AgNO_3_ precursor solution was prepared by dissolving 0.034 g of silver nitrate (AgNO_3_) in 200 mL of distilled water and then placed on a magnetic stirrer at 150 rpm (steady stirring), with the anticipated color alteration. 5–6 (15 mL) drops of the *Pistacia lentiscus* plant extract solution were added to the silver nitrate solution using a plastic pipette at various time intervals, with continuous stirring. Following the 5-minute interval drops, 5 mL of the solution was collected from the flask at 10 min, 15 min, 30 min, 60 min, and 90 min. They were transferred to an empty centrifuge tube for UV analysis, and the remaining solution of silver nitrate + plant extract was left on the magnetic stirrer, with continuous stirring for a total of 2 h.

Thereafter, the *Pistacia lentiscus* mixture (AgNO_3_ + distilled water + *Pistacia lentiscus*) in the flask was centrifuged at 4500 rpm (25 °C) for 30 minutes and dried in an oven at 35 °C for 48 h. The derived crystalline particles from the oven were kept in an Eppendorf tube for further use in the study. As shown in Fig. 4, an identical technique was implemented for the *Arbutus andrachne* solution to synthesize *Arbutus andrachne* silver nanoparticles. The isolated particles were positioned individually in a vacant centrifuge tube.

**Fig. 4:**
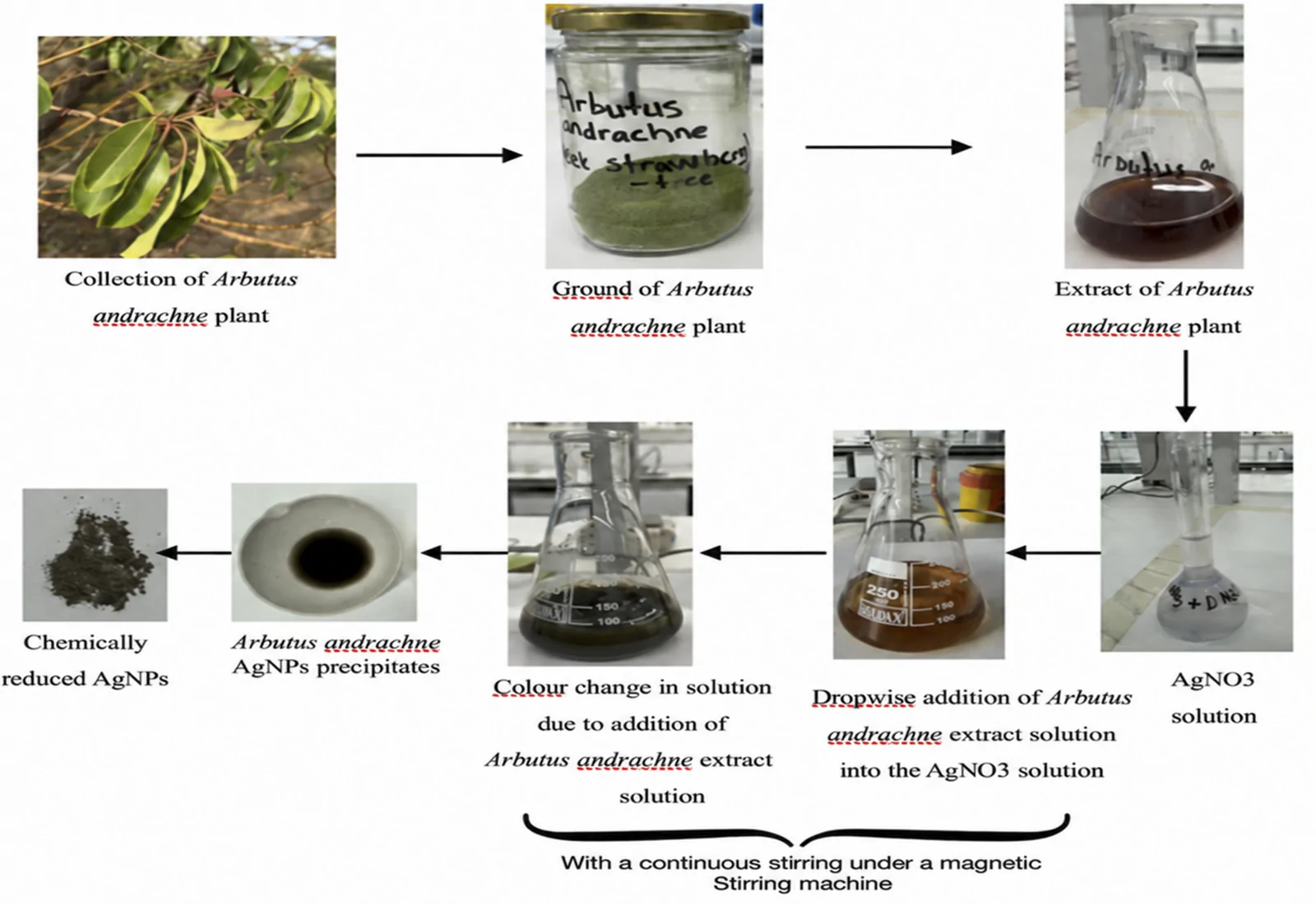
*Arbutus andrachne* silver nanoparticle synthesis.

### 2.5 Gas chromatography-mass spectrometry (GC-MS)

GC-MS, along with the internal WILEY library, was used to analyze the biochemical constituents of the plant extract, following the methodology outlined by Kumar, V. *et al.*^6^ with few modifications. The TRB-5MS column was run in electron impact mode at 70 eV. The carrier gas used was 99.99% helium, which flowed continuously at a rate of 1 mL min^−1^. The split volume of the injection was 0.5 ET, with an injection temperature of 180 °C, and the transfer line temperature was maintained at 250 °C. The temperature of the system oven was 50 °C and was increased at a rate of 7 °C per minute. The mass spectrum data were examined at 70 eV with 0.5 s scan intervals, covering a full mass scan that ranged from 25 *m*/*z* to 1000 *m*/*z*. By comparing the retention indices and matching the recorded mass spectra of each compound to the Wiley MS libraries and NIST 14 mass spectral library of chemicals, the identifications of the plant extract components were performed.

### 2.6 UV-vis spectrophotometer

Optical absorbance was measured utilizing a Shimadzu brand UV-2450 model (UV-vis spectrophotometer). This instrument was used to observe the synthesis of AgNPs at various time intervals. The absorbance was measured at different time intervals of 5, 10, 15, 30, 60, and 90 min. UV-visible spectrophotometer wavelength range of 300–700 nm validated the synthesis of AgNPs. During the reaction, Ag^+^ from silver nitrate was reduced to AgNP, which can be tracked by watching the peak absorbance and the accompanying color change. The alterations in colour of the AgNP solution from colorless to dark brown in 90 min confirmed the formation of AgNPs. The optical absorption of the prepared AgNPs was examined by UV-vis spectroscopy. Spectral measurements were collected between 300 and 700 nm using a cuvette, with a 1 cm light path.

### 2.7 Silver nanoparticles characterization methods

Scanning electron microscopy (SEM), transmission electron microscopy (TEM), X-ray diffraction (XRD) and energy-dispersive X-ray (EDX) were used to analyze the AgNPs. A field-emission scanning electron microscope (Zeiss Gemini 500, Oberkochen, Germany) was used to obtain EDX spectra and electron microscopy images. With the aid of a Cu Kα anode (*λ* = 0.1542 nm) on a Bruker D8 Advance X-ray diffractometer operating at 40 kV and 30 mA, the XRD patterns were recorded. The morphology and topography of the synthesized AgNPs were determined by TEM (JEOL JEM-1011).

### 2.8 Diphenyl-2-picrylhydrazyl (DPPH) radical scavenging assay

0.0197 g of DPPH was introduced into an empty flask, followed by the addition of 50 mL of methanol. 0.1 g of *Pistacia lentiscus* M extract and 0.1 g of *Pistacia lentiscus* E extract were each placed in separate centrifuge tubes, followed by the addition of 10 mL of methanol to both tubes. An identical procedure was implemented for *Arbutus* M, *Arbutus* E, *Arbutus* AgNPs, and *Pistacia lentiscus* AgNPs. Subsequently, 0.1 g of ascorbic acid was combined with 10 mL of methanol in a separate tube. Sample concentrations of 10, 20, 50, 100, 150, and 200 mg mL^−1^ were introduced into separate test tubes, as indicated in the Table 1 mL of the prepared DPPH mixture was added to each test tube, and they were kept in the dark for 30 minutes. The absorbance of DPPH (control) and all the samples was measured using UV-vis spectrometry.

**Table 1:** GC-MS results of the *P. lentiscus* ethanol and methanol extracts

No	RT	Name of compounds	Molecular formula	Molecular mass (g mol^−1^)	Peak area (%)	Compound nature	Biological activity
**Ethanol extract**							
1	3.501	Acetic acid	CH_3_COOH	60.05	2.77	Ethanoic acid	Antimicrobial
2	4.325	Benzoyl bromide	C_7_H_7_Br	171.04	3.55	Benzyl	Antibacterial and antifungal
3	5.172	Ethyl orthoformate	CH(OC)_3_	148.20	1.57	Ether	Antibacterial
4	11.561	4H-pyran-4-one	C_5_H_4_O_2_	96.08	1.61	Unsaturated cyclic chemical compound	Antimicrobial
5	12.619	1-Dodecene	C_12_H_24_	168.32	1.28	Alkene	Antimicrobial, antifungal and antitumor
6	16.035	1,2,3-Benzenetriol	C_6_H_6_O_3_	126.11	6.05	Hydroxy	Antioxidant, antibacterial and anti-aging
7	16.565	1-Tetradecene	C_14_H_28_	196.37	2.03	Alkene	Antibacterial and antifungal
8	18.134	3-Hexanone	C_6_H_12_O	100.16	1.99	Ketone	Antibacterial
9	18.890	Carbamic acid	CH_3_NO_2_	61.04	1.98	Amine and carboxylic acid	Antibacterial
10	19.337	3,4-Dimenthylcylohexanol	C_8_H_16_O	128.21	1.80	Cyclohexanol	Not yet recorded
11	20.123	Cetene	C_16_H_32_	224.45	1.88	Alkane	Antimicrobial
12	20.486	1,3,4,5-Tetrahydroxycyclohexanecarboxylic acid	C_7_H_12_O_6_	192.17	5.65	Ethanol	Antimicrobial
13	20.529	Butyric acid	C_4_H_8_O_2_	88.11	3.21	Carboxylic acid	Antimicrobial
14	20.551	4(1H)-pyrimidinone	C_4_H_4_N_2_O	96.09	3.72	Pyrimidine	Antibacterial and antifungal
15	20.331	1-Octadecene	C_18_H_36_	252.50	1.25	Alkene	Antioxidant and antibacterial
16	28.529	9,12,15-Octadecatrienoic acid	C_18_H_30_O_2_	278.40	3.77	Methyl ester	Anti-inflammatory and antimicrobial
17	34.919	Octadecatrienoic acid	C_18_H_36_O_2_	284.50	3.00	Fatty acid	Antitumor
18	35.152	1,4-Benzenedicarboxylic acid	C_8_H_6_O_4_	166.13	3.15	Carboxy groups	Anti-microbial
19	36.512	Tocospiro	C_29_H_50_O_4_	462.7	1.29	Not yet recorded	Antimicrobial, antioxidant and antitumor

**Methanol extract**							
1	3.48	Acetic acid	CH_3_COOH	60.05	5.22	Ethanoic acid	Antimicrobial
2	3.71	Butanoic acid	C_4_H_8_O_2_	88.11	3.43	Carboxylic acid	Antimicrobial and anticarcinogenic
3	3.95	Silanediol	H_6_O_4_Si	64.116	6.33	Hydroxyl	Antitumor
4	4.06	Acetic acid	CH_3_COOH	60.05	1.49	Ethanoic acid	Antimicrobial
5	4.32	Diglycerol	C_6_H_14_O_5_	166.17	1.66	Glyceride	Antimicrobial
6	4.36	Pyridine	C_5_H_5_N	79.1	1.82	Heterocyclic	Antimicrobial and antiviral
7	6.51	Oxime	CHNO_2_	43.024	8.01	Imines	Anti-inflammatory and
8	7.77	Pyridine	C_5_H_5_N	79.1	1.09	Heterocyclic	Antimicrobial
9	8.08	Phenol	C_6_H_6_O	94.11	4.3	Acidic	Antiseptic
10	8.17	Cyclotetrasiloxane	H_8_O_4_Si_4_	184.4	1.49	Methyl	Antibacterial
11	11.45	Cyclopentasiloxane	(CH_3_)_2_SiO_5_	370.77	1.23	Organosilicon	Antioxidant
12	12.55	Catechol	C_6_H_6_O_2_	110.11	2.02	Benzene	Anticancer
13	13.01	Leucylglycine	C_8_H_16_N_2_O_3_	188.22	4.17	Dipeptide	Antioxidant
14	13.25	Cyclopentadione	C_9_H_14_O_2_	154.21	14.78	Cyclic	Antimicrobial
15	14.11	Hydroquinone	C_6_H_6_O_2_	110.11	7.76	Aromatic	Antioxidative
16	14.43	Guanidine	CH_5_N_3_	59.07	1.31	Nitrogen	Antitumor
17	18.61	2,4-Di-*tert*-butylphenol	C_14_H_22_O	206.32	1.42	Phenol	Antifungal
18	18.85	Ethylbenzonitrile	C_9_H_9_N	131.18	2.82	Aromatic nitrile	Not recorded
19	20.30	Urea	CH_4_N_2_O	60.056	1.69	Acidic	Antibacterial
20	28.12	Oleic acid	C_18_H_34_O_2_	282.5	8.04	Fatty acid	Antibacterial
21	28.18	9-Octadecenoic acid	C_18_H_34_O_2_	282.5	3.22	Monounsaturated fatty acid	Antioxidant

### 2.9 Antimicrobial study

#### 2.9.1 Agar preparation

A prepared 500 mL Nutrient agar solution was decanted into a petri plate. A burner was employed to maintain sterility in the surroundings during this period. Then, the solution in the plate was allowed to cool for about 20 min; afterward, the agar plates were kept in the refrigerator for further use. Then, the prepared nutrient broth solution was dispensed into two separate centrifuge tubes. *Escherichia coli* (*E. coli*) was introduced into one tube, while *Enterococcus faecalis* (*E. faecalis*) was introduced into the other, and the tubes were labeled accordingly; they were subsequently incubated at 36.5 °C for 24 h to facilitate growth of the bacteria.^7^

#### 2.9.2 Antibacterial study

For the concentration of previously prepared treatments (plant and nanoparticles), AE, AM, PE, PM, *A. andrachne* AgNPs (A.A AgNPs), and *P. lentiscus* AgNPs (P.L AgNPs), 0.05 g of AE, AM, PE, and PM were dissolved in separate test tubes containing 10 mL of distilled water as a stock. Furthermore, 0.05 g of A.A AgNPs and P.L AgNPs were also dissolved in separate test tubes containing 10 mL of DMSO as a stock. Different concentrations (100 µg mL^−1^, 200 µg mL^−1^, and 300 µg mL^−1^) of the stock were prepared in separate tubes for the treatment, and 0.5 mL of DMSO was incorporated into the P.L AgNP and A.A AgNP solutions. Petri dishes were labeled accordingly, and three replicates were conducted for each petri dish; then, the average of the results was calculated. Petri dishes were inoculated with the *P. lentiscus* AgNP solution (AZI, 100 µg mL^−1^, 200 µg mL^−1^, and 300 µg mL^−1^) and two different bacterial strains (*E. coli* and *E faecalis*), and an identical technique was implemented for the A.A AgNP solution. The AE, AM, PE, and PM solutions and DW (distilled water) were cultured with the bacterial strains, and commercially prepared azithromycin (AZI 15 µg) discs were used as antibiotics in this experiment. The discs were used directly without further preparation or dilution, according to the manufacturer's instructions. A burner was used to maintain a sterile environment during this procedure. Bacterial strains were cultured in designated petri dishes using swabs. The pre-prepared tiny circular filter papers were meticulously positioned in each compartment of the petri dishes. Various extracts with different concentrations were applied onto circular filter sheets utilizing a micropipette. The prepared petri dishes were incubated, and a vernier caliper was used to measure the zone of inhibition.^8–12^

### 2.10 Proliferation and cytotoxicity assay

The cytotoxicity of AgNPs produced from *Arbutus andrachne* (A.A AgNPs) and *Pistacia lentiscus* (P.L AgNPs) was investigated in MDA-MB-231 breast cancer cells by the trypan blue exclusion method. Approval for the use of the MDA-MB-231 cell line was obtained from the Biotechnology Research Centre Ethical Committee (BRCEC2011-01). The cells were introduced into 96-well plates at an initial density of approximately 1 × 10^5^ cells/mL and cultured for 24 h before nanoparticle treatment. After this period, the cells were exposed to either A.A AgNPs or P.L AgNPs at 0, 50, 250, and 500 µg mL^−1^ and maintained for 48 h. Cells cultured in DMEM without nanoparticle treatment were used as the control. At the end of the exposure period, the cells were collected and combined with a 0.4% trypan blue solution. Following 10 min of dye exposure, viable and non-viable cells were differentiated according to dye uptake, with viable cells remaining unstained, while non-viable cells appeared blue. The cells inside the plates were then counted from 30 random locations under an inverted microscope (Leica DFC295, Leica Microsystems, Germany) for the determination of the percentage viability of cells. The experiments were conducted in triplicate (*n* = 3). The percentage viability of cells was obtained by utilizing the following equation:
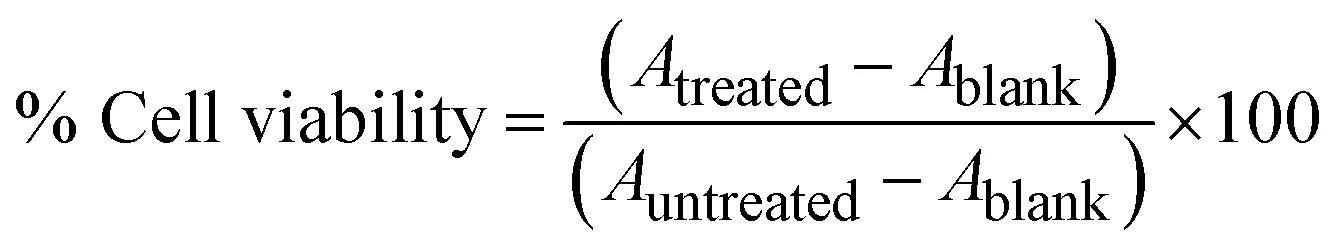


### 2.11 Statistical analysis

All the experiments were conducted at least three times (*n* ≥ 3). All the data presented in the results were expressed as the standard deviations from the mean values (mean ± standard deviation). One-way and two-way ANOVA tests were carried out to evaluate all experimental data.

## 3. Result and discussion

### 3.1 Gas chromatography mass spectrometry (GC-MS)

The GC-MS results from the analysis of the plant extract are presented in Tables 1 and 2, with their chromatogram presented in Fig. 5 and 6. Tables 1 and 2 present the chemical compounds, retention times, and reported biological properties of the *P. lentiscus* and *A. andrachne* extracts. The *P. lentiscus* ethanol extract had 19 chemical components, yielding a total peak area of 83.30. *P. lentiscus* ethanol exhibited a significant concentration of benzenetriol, characterized by a peak area of 6.05%, whereas octadecene displayed a minimal peak area at 1.25% of the overall chemical composition. Benzenetriol exhibited antioxidant, antimicrobial, and anti-aging properties. Octadecene exhibited antioxidant and antibacterial effects.

GC-MS results of the *A. andrachne* ethanol and methanol extracts

**Table:** 

No	RT	Name of the compound	Molecular formula	Molecular mass	Peak area (%)	Compound nature	Biological activity
Ethanol extract							
1	3.54	Acetic acid	C_2_H_4_O_2_	60.05	3.17	Ethanoic acid	Antimicrobial
2	4.34	6-Benzylthiobenzoyl bromide	C_7_H_5_BrO	185.02	2.45	Benzyl	Antimicrobial
3	4.96	Propanoic acid	C_3_H_6_O_2_	74.08	1.07	Carboxylic acid	Antifungal
4	5.18	Ethyl orthoformate	C_4_H_10_O_3_	148.20	3.21	Formic acid	No activity recorded
5	6.506	1,3-Dihydroxyacetone dimer	C_6_H_12_O_6_	180.16	1.57	Nitric acid ester	No activity recorded
6	12.62	1-Dodecene	C_12_H_24_	168.32	1.23	Alkene	Antimicrobial and antifungal
7	14.12	Hydroquinone	C_6_H_6_O_2_	110.11	2.68	Phenol	Antioxidative
8	16.57	2-Tetradecene	C_14_H_28_	196.37	2.29	Hydrocarbon	Antioxidant
9	16.72	Tetradecane	C_14_H_30_	198.39	2.79	Hydrocarbon	Antibacterial and antifungal
10	17.58	1,3-Propanediol	C_3_H_8_O_2_	76.09	2.87	Organic compound	Antifreeze
11	20.12	Cetene	C_16_H_32_	224.43	3.64	Alkene	Antibacterial and antifungal
12	20.25	Hexadecane	C_16_H_34_	226.44	1.14	Alkane hydrocarbon	Antibacterial and antioxidant
13	20.51	Amylcycloheptanone	C_11_H_20_O	210.35	4.91	Cyclic ketone	No activity recorded
14	20.61	Nonanoic acid	C_9_H_18_O_2_	158.24	8.00	Fatty acid	Antifungal and antimicrobial
15	23.33	1-Octadecene	C_18_H_36_	252.49	1.25	Hydrocarbon and alkene	Antioxidant
16	27.79	2-Hexadecen-1-ol	C_16_H_32_O	240.42	2.86	Chlorophyll	Antimicrobial
17	34.92	Octadecanoic acid	C_18_H_36_O_2_	284.50	2.92	Saturated fatty acids	Antitumor
18	35.16	1,4-Benzenedicarboxylic acid	C_8_H_6_O_4_	166.13	2.88	Monobutyl ester	No activity recorded

**Table:** 

No	RT	Name of the compound	Molecular formula	Molecular nature	Peak area (%)	Compound nature	Biological activity
Methanol extract							
1	3.46	Acetic acid	C_2_H_4_O_2_	60.05	6.72	Ethanoic acid	Antimicrobial
2	3.82	2-Propanone	C_3_H_6_O	58.08	5.8	Ketone	Antimicrobial and anticancer
3	3.92	Silanediol	SiH_4_O_2_	64.12	3.9	Organosilicon	Not yet reported
4	4.29	Propanoic acid	C_3_H_6_O_2_	74.08	6.76	Carboxylic acid	Antioxidant, antitumor, and antimicrobial
5	4.33	1,4-Dioxane	C_4_H_8_O_2_	88.11	2.19	Ether	Antimicrobial
6	4.69	Sulfurous acid	H_2_SO_3_	82.07	1.41	Sulfur oxoacid	Antimicrobial
7	5.43	Pentane	C_5_H_12_	72.15	1.67	Alkane	Toxicity
8	8.09	Phenol	C_6_H_6_O	94.11	2.26	Acidic	Antiseptic
9	8.32	2-Hydroxy-gamma-butyrolactone	C_4_H6O3	102.09	2.49	Hygroscopic	Antibiotic, antifungal, and antitumor
10	11.56	4H-pyran-4-one	C_5_H_4_O_2_	96.08	1.15	Unsaturated cyclic chemical	Antimicrobial and antifungal
11	12.53	Catechol	C_6_H_6_O_2_	110.1	1.64	Benzene	Anti-cancer, anti-inflammatory, and antimicrobial
12	13.03	Dianhydromannitol	C_6_H_10_O_4_		2.11	Ethanol	Analgesic and anti-inflammatory
13	13.26	1-(5-Methyl-2-thienyl)-1-propanone	C_8_H_10_OS	154.23	2.67	Ketone	Antimicrobial and antitumor
14	14.12	Hydroquinone	C_6_H_6_O_2_	110.11	9.73	Aromatic organic	Antioxidative and neuroprotective
15	16.04	1,2,3-Benzenetriol	C_6_H_6_O_3_	126.11	2.03	Polyphenol	Antioxidant and antibacterial
16	19.57	β-d-Glucopyranoside	C_6_H_12_O_6_	180.16	1.5	Methyl	Antioxidant and anti-inflammatory
17	19.63	α-d-Galactopyranoside	C_6_H_14_O_6_	194.18	1.63	Methyl	Antibacterial and antioxidant
18	19.75	Methyl-β-D-thiogalactoside	C_7_H_14_O_5_S	210.25	3.06	O-glycosyl	Antibacterial and antifungal
19	20.35	Butyraldehyde	C_4_H_8_O	72.11	1.48	Butanal	Antimicrobial
20	20.42	4(1H)-pyrimidinone	C_4_H_4_N_2_O	96.09	1.02	Heterocyclic	Antibacterial and antifungal
21	33.01	Lupeol	C_30_H_50_O	426.70	7.91	Hydroxy group	Anti-inflammatory and cancer preventive

**Fig. 5:**
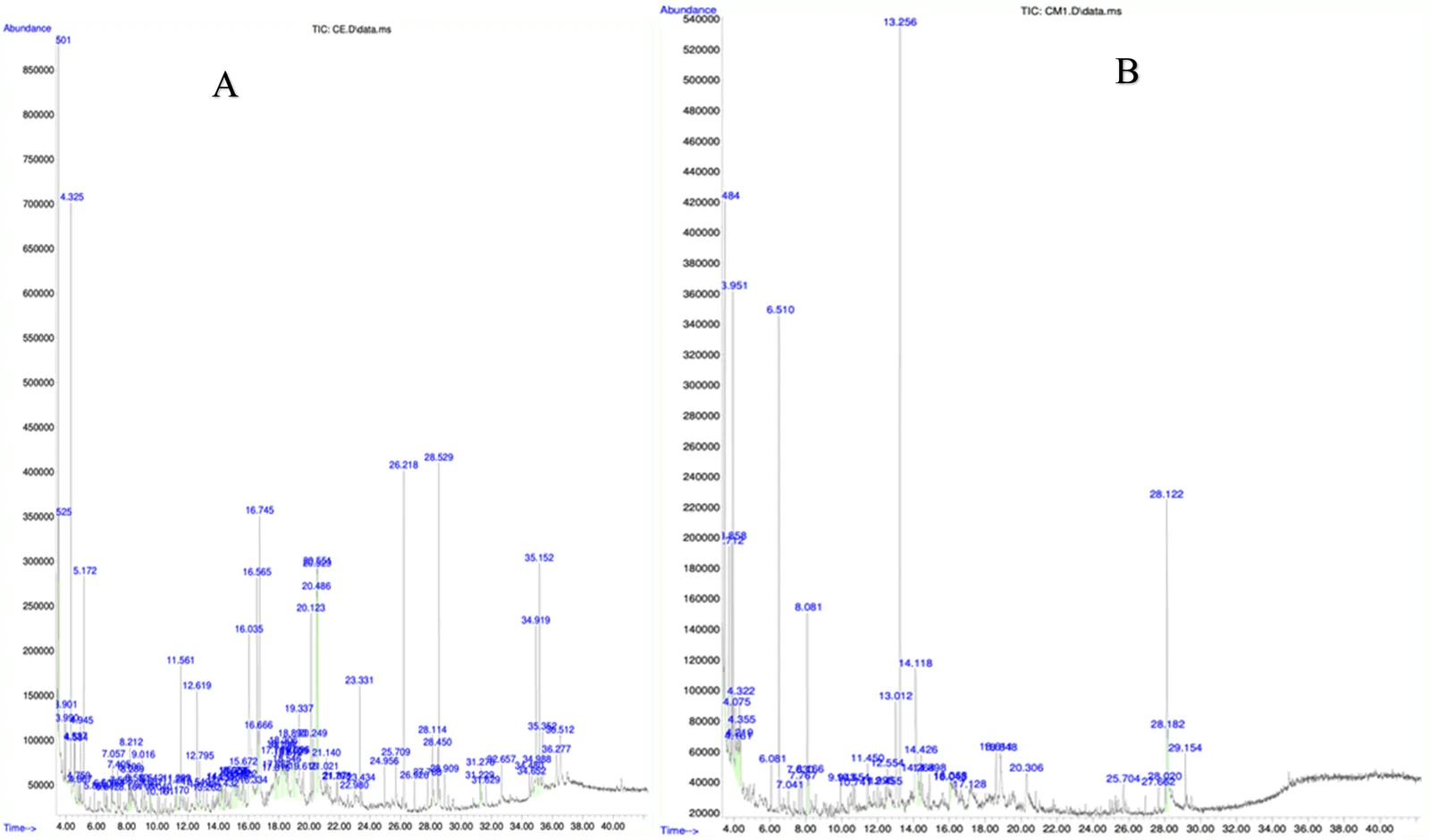
GC-MS graphs of *Pistacia lentiscus* (A) ethanol and (B) methanol extracts.

**Fig. 6:**
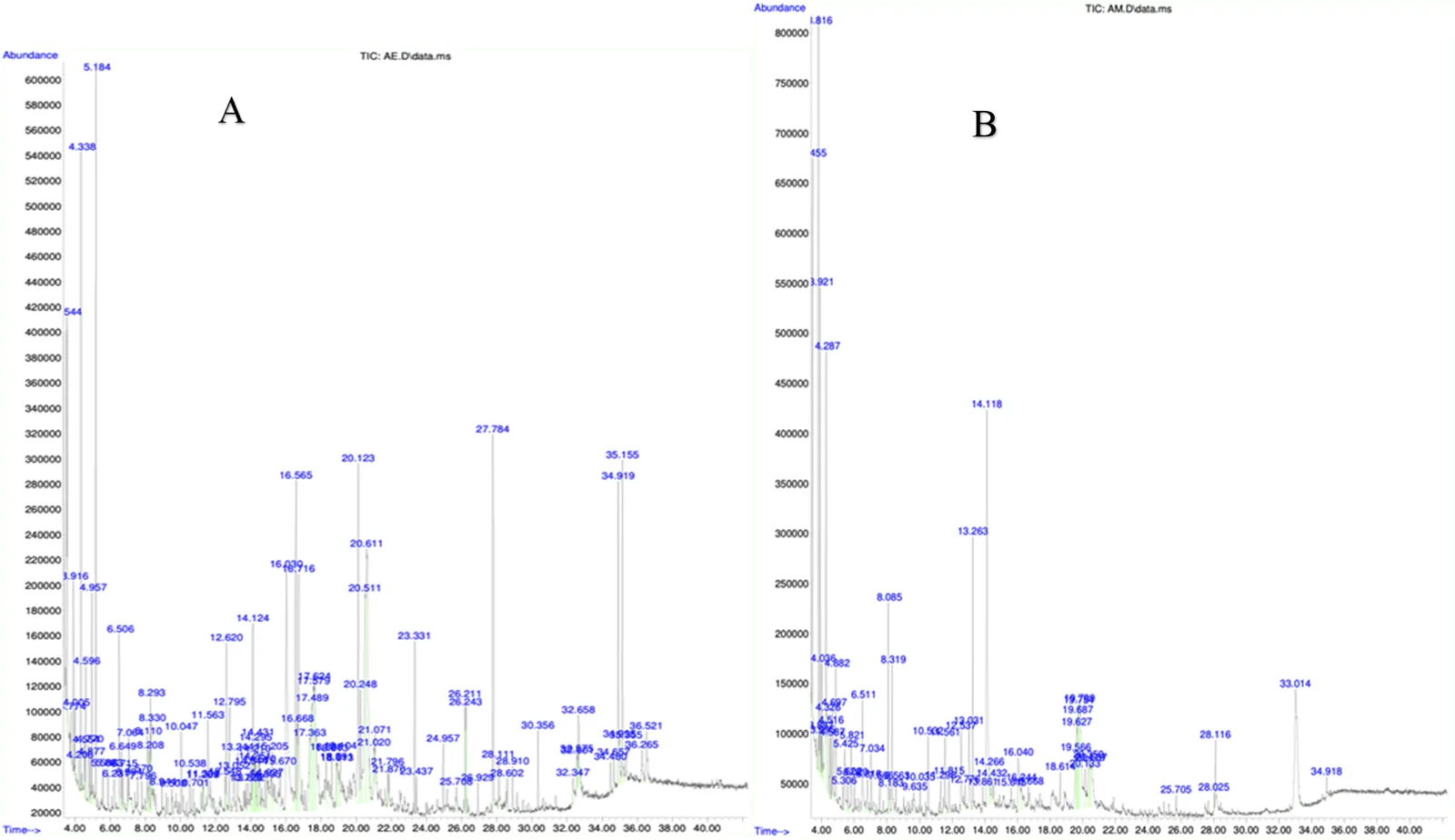
GC-MS analysis of the *Arbutus andrachne* (A) ethanol and (B) methanol extracts.

Table 1 presents the chemical compounds, retention times, and the biological properties of the *P. lentiscus* extract. In Table 1, it is shown that the *P. lentiscus* Methanol extract has 21 chemical compounds. *Pistacia* E exhibited a substantial concentration of cyclopentadione, with a peak area of 14.78%, whereas pyridine demonstrated the lowest peak area at 1.09% of the overall chemical composition. Cyclopentadione exhibited antioxidant and antibacterial properties, while Pyridine exhibited antibacterial effects. The GC-MS results obtained in this study aligned with those reported by Kilic-Buyukkurt *et al.*,^13^ indicating consistency in the chemical composition.

Table 2 presents the chemical compounds, retention times, and reported biological properties of the *A. andrachne* extracts. As illustrated in Table 2, the *Arbutus andrachne* methanol extract contains a significant concentration of nonanoic acid, exhibiting an 8% peak area, whereas propanoic acid demonstrates the lowest peak area at 1.07% of the overall chemical composition. Nonanoic acid exhibited antifungal and antibacterial properties. Propanoic acid exhibited antifungal effects. In addition, as shown in Table 2, the *Arbutus andrachne* ethanol extract contains a significant concentration of hydroquinone, exhibiting a peak area of 9.73%, whereas pyrimidinone demonstrates the lowest peak area at 1.02% of the overall chemical composition. Hydroquinone exhibited antioxidative, neuroprotective, and immunomodulatory properties. In addition, pyrimidinone exhibited antibacterial, anticancer and anti-inflammatory properties.^14,15^

The phytochemical constituents detected in the plant extracts could have played an important role during nanoparticle synthesis. Compounds such as hydroquinone and benzenetriol possess hydroxyl functional groups capable of participating in electron-transfer reactions, which may support the conversion of Ag^+^ into Ag^0^ during AgNP formation. Following reduction, some of these plant-derived molecules may remain associated with the nanoparticle surface, providing a protective layer that could reduce particle aggregation and improve stability. Moreover, phytochemicals with known antioxidant or antimicrobial properties associated with the AgNP surface could potentially influence the biological response of the resulting nanoparticles. Thus, the antibacterial and anticancer effects recorded in this study may reflect contributions from both the silver nanoparticles and surface-associated plant constituents. Nevertheless, the present findings do not establish the activity of individual phytochemicals or demonstrate a synergistic interaction. Therefore, further mechanistic studies are needed to determine the specific roles of these compounds in AgNP formation, stabilization, and biological activity.

### 3.2 UV-vis spectrophotometer

The solution of AgNPs was analyzed using UV-vis spectroscopy at a wavelength range of 200–800 nm, which confirmed the formation of AgNPs. The graph (Fig. 7) illustrated that absorbance increased with the prolonged incubation (0 min, 5 min, 10 min, 15 min, 30 min, 60 min, and 90 min) of silver nitrate and plant extract.

**Fig. 7:**
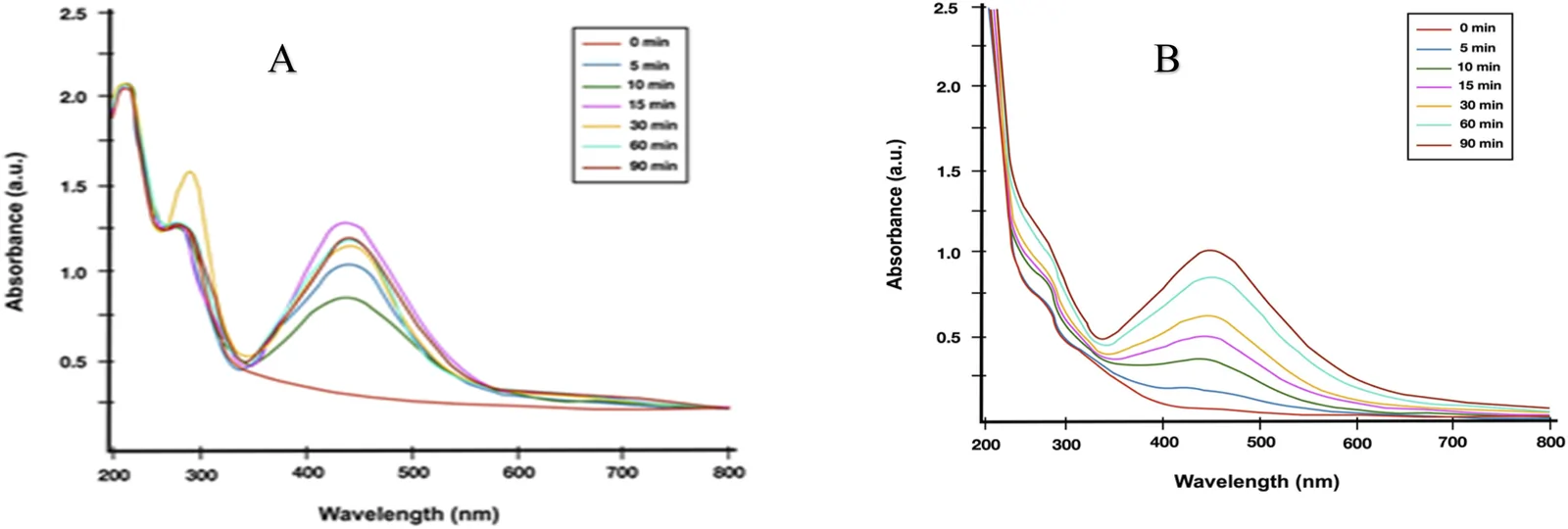
UV results of the (A) *Arbutus andrachne* plant extract and (B) *Pistacia lentiscus* plant extract.

The observation of the reaction, as indicated by the color change in the mixture of *Arbutus andrachne*, *Pistacia lentiscus* leaf extract, and silver nitrate solution, was facilitated using a UV-vis spectrophotometer. The surface plasmon resonance (SPR) of *Arbutus andrachne* and *Pistacia lentiscus* AgNPs generated throughout the reaction period resulted in a color transition from bright yellow to deep brown. Samples collected at 0, 5, 10, 15, 30, 60, and 90 min intervals were analyzed to identify the maximum wavelength within the 200–800 nm range. The maximal wavelength of the synthesized AgNPs was found to be 450 nm. Peak absorbance, which occurred at 450 nm, revealed the presence of AgNPs.^16^ The value aligned with the maximum absorbance readings for AgNPs derived from the plant extracts of *Gymnema sylvestre* (442 nm),^17^*Calophyllum tomentosum* (438 nm),^18^*Cocos nucifera* (380 nm),^19^ and *Cicer arietinum*.^20^

### 3.3 Zeta potential measurements

Fig. 8 illustrates the zeta potential results of *Arbutus andrachne*. The average peak-1 of aqueous A.A AgNPs at 112.2 nm was found to be 100%, with an average zeta distribution of 90.87 nm, signifying high-quality results. The zeta potential value was recorded to be −16.14 mV, which signified the stability of the nanoparticles. The negative value was an indication of nanoparticle stability and agglomeration inhibition. The results aligned with those obtained for silver nanoparticles synthesized from leaf extracts of *F. religiosa* and *C. haematocephala*.^21,22^ The capping effect of biomolecules from the leaf extract of *Arbutus andrachne* resulted in a negative potential value.

**Fig. 8:**
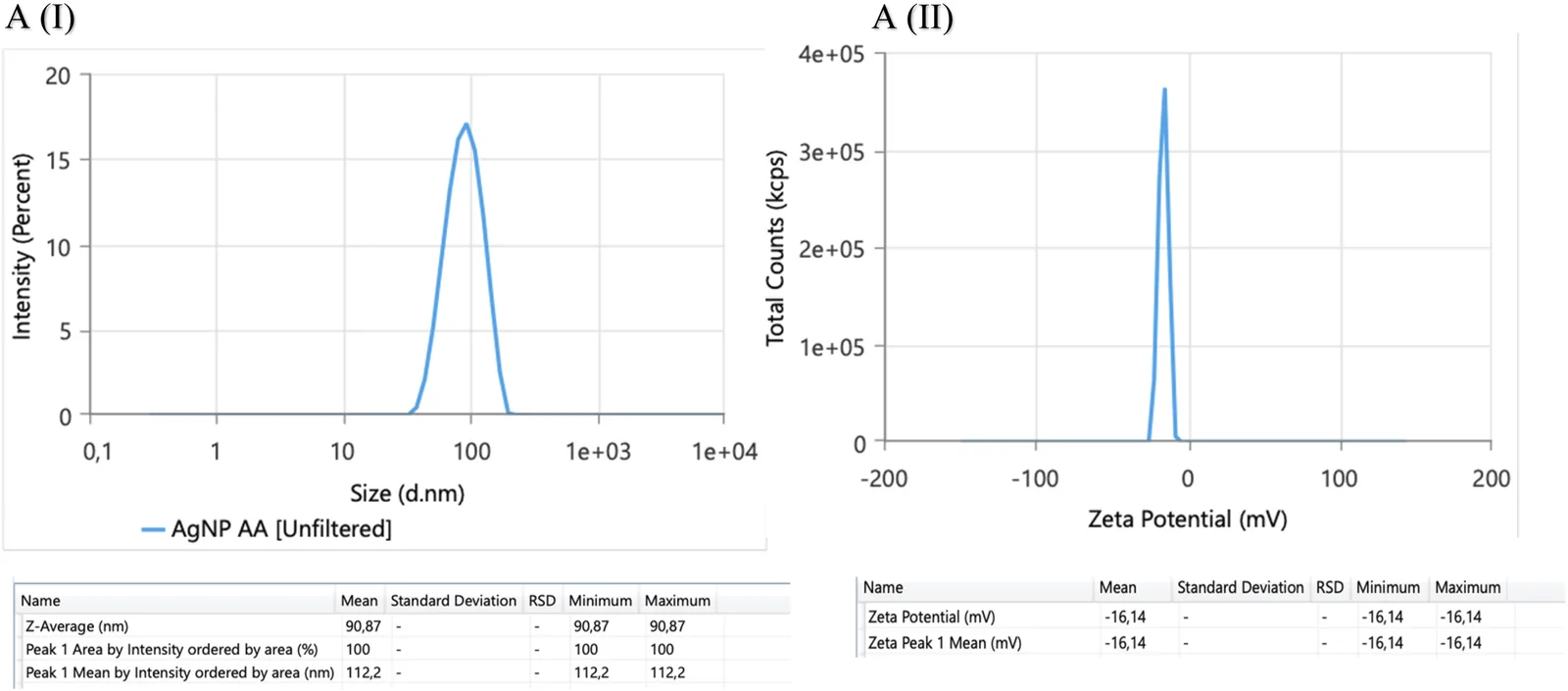
Zeta potential and particle size distributions of the AgNPs synthesized using the leaf extract of *Arbutus andrachne*, where A (I) indicates the size and A (II) indicates the zeta potential.

Fig. 9 confirmed that the aqueous *P. lentiscus* AgNPs exhibited an average zeta distribution of 78.26 nm, with 85.23% of the average peak-1 at 123.2 nm, indicating high-quality results. The zeta potential value was measured at −22.66 mV, indicating the charge stabilization of the nanoparticles. The negative potential value demonstrated by the biosynthesized AgNPs indicated the existence of bioorganic constituents in the extract, serving as capping agents.^23^ The result aligned with the AgNPs synthesized from *E. camaldulensis* and *T. arjuna* plant extracts.^24^

**Fig. 9:**
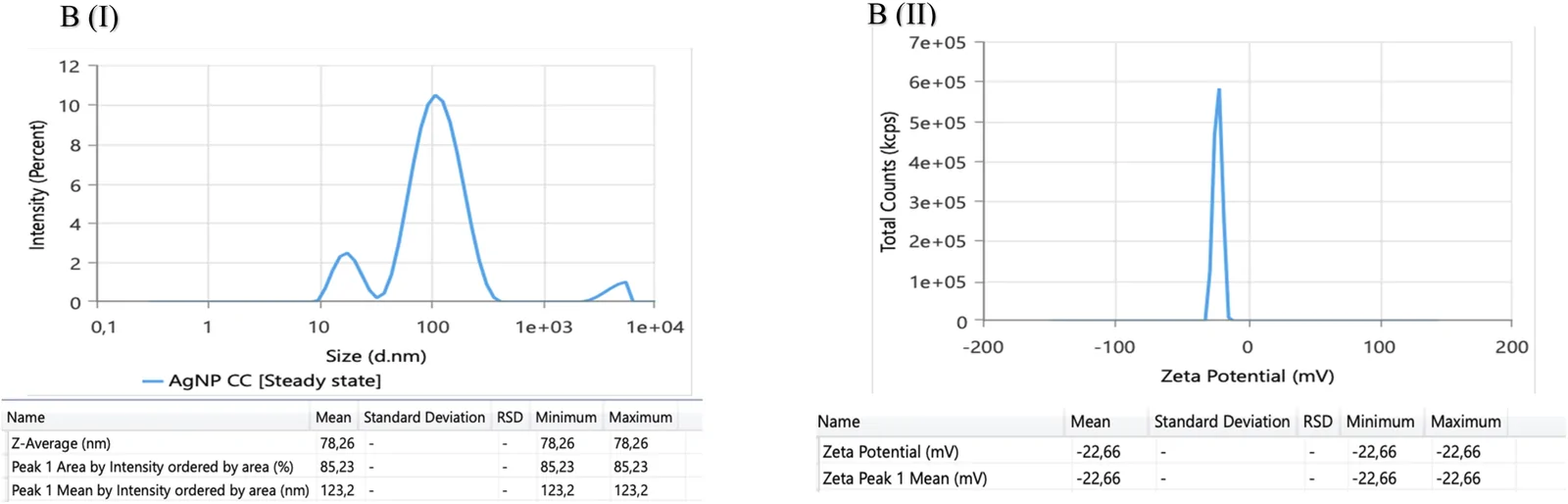
Zeta potential and particle size distributions of the AgNPs synthesized using the leaf extract of *Pistacia lentiscus*, where B (I) indicates the size and B (II) indicates the zeta potential.

### 3.4 Scanning electron microscopy (SEM)

Scanning electron microscopy (SEM) was performed to examine the surface shape and dimensions of the synthesized silver nanoparticles (AgNPs). Representative SEM pictures of silver nanoparticles generated from the *Arbutus andrachne* and *Pistacia lentiscus* plant extracts in this study are displayed in Fig. 10 at varying magnification scales. Fig. 10A illustrates that the biosynthesized AgNPs generally exhibit a spherical morphology, while Fig. 10B demonstrates increased aggregation attributable to the sticky characteristics of the synthesized AgNPs from the *Arbutus andrachne* extract. As depicted in Fig. 10A, the aggregation of AgNPs is evident in the SEM images, and the nanoparticles exhibit a mostly uniform morphology with a narrow size distribution. In contrast, Fig. 10B illustrates excessive clustering among small, uniform clusters, with a narrow size distribution. The observed sizes of the agglomerated nanoparticles for *Arbutus andrachne* AgNPs and *Pistacia lentiscus* AgNPs ranged from 15 to 40 nm and 40 to 120 nm, respectively, exhibiting polydispersity. Shyam P. *et al.* reported the fabrication of spherical AgNPs with a typical size of 51–230 nm using *Saraca indica* plant extract.^25^ Additionally, Swati J. and Anita G. reported the biosynthesis of AgNPs, which ranged in size from 10 to 35 nm, employing *Citrus reticulata* peel extract.^26^ Literature reports indicate that silver nanoparticles have been synthesized from multiple plant extracts and can exhibit diverse morphologies, including spherical, hexagonal, and triangular shapes, contingent upon the chemical composition and the plant extract concentration.^27^

**Fig. 10:**
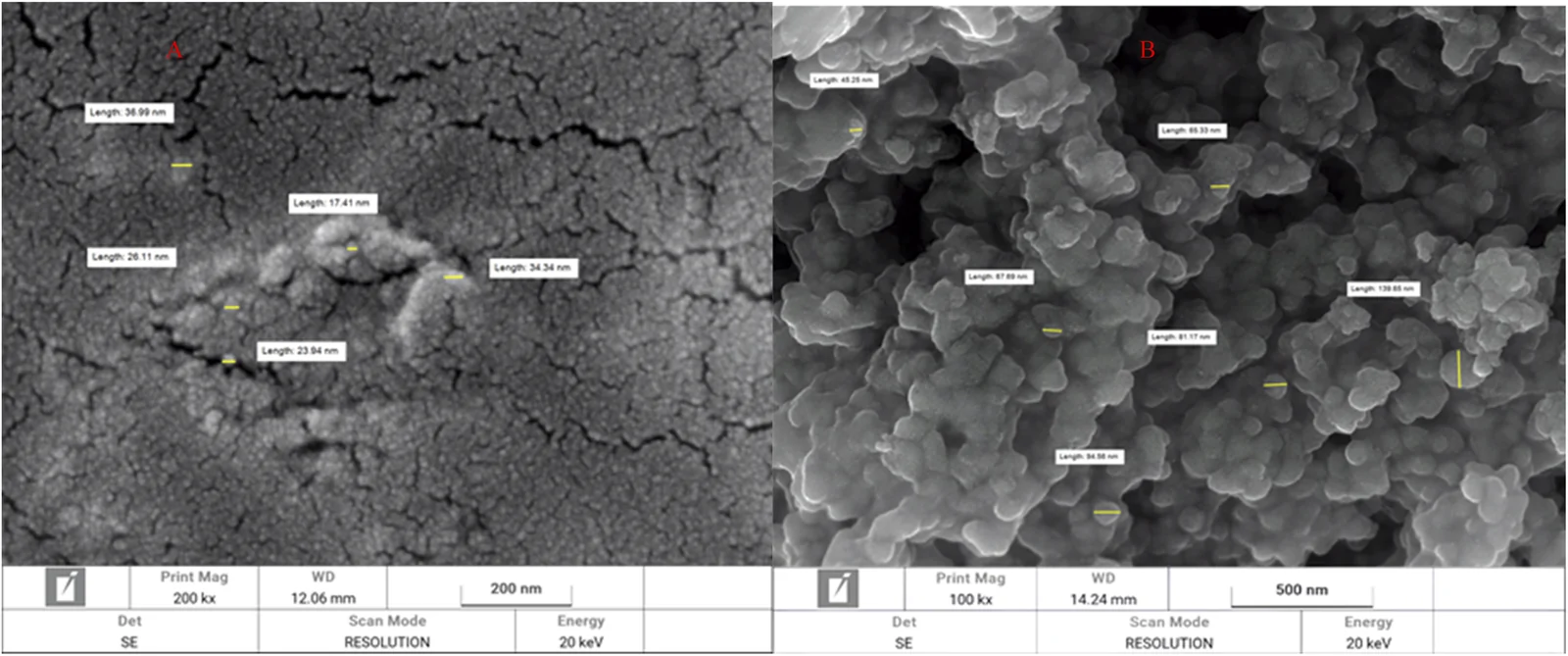
SEM images of the synthesized AgNPs: (A) *Pistacia lentiscus* AgNPs and (B) *Arbutus andrachne* AgNPs.

### 3.5 Transmission electron microscopy (TEM)

The size and morphological properties of AgNPs were examined by transmission electron microscopy. The TEM picture depicted in Fig. 11 clearly illustrates that the AgNPs possess a spherical morphology. The image displays clusters of tiny grains and several dispersed nanoparticles, supporting the findings acquired using SEM and TEM.^28^ The AgNPs produced by *Arbutus andrachne* and *Pistacia lentiscus* were measured to be 60 nm, with some smaller particles exhibiting a spherical morphology, as illustrated in Fig. 11A and B. Similar results were documented concerning the fabrication of nanoparticles utilizing the fruit extract of *Actinidia deliciosa*. It was reported that the negatively charged carboxylic groups in proteins interacted electrostatically with the AgNPs, and the free amino groups and cysteine residues in proteins attached to the nanoparticles. Consequently, they serve a significant function as stabilizing agents and for natural sealing. Nonetheless, the agglomeration of the nanoparticles was induced.^29,30^

**Fig. 11:**
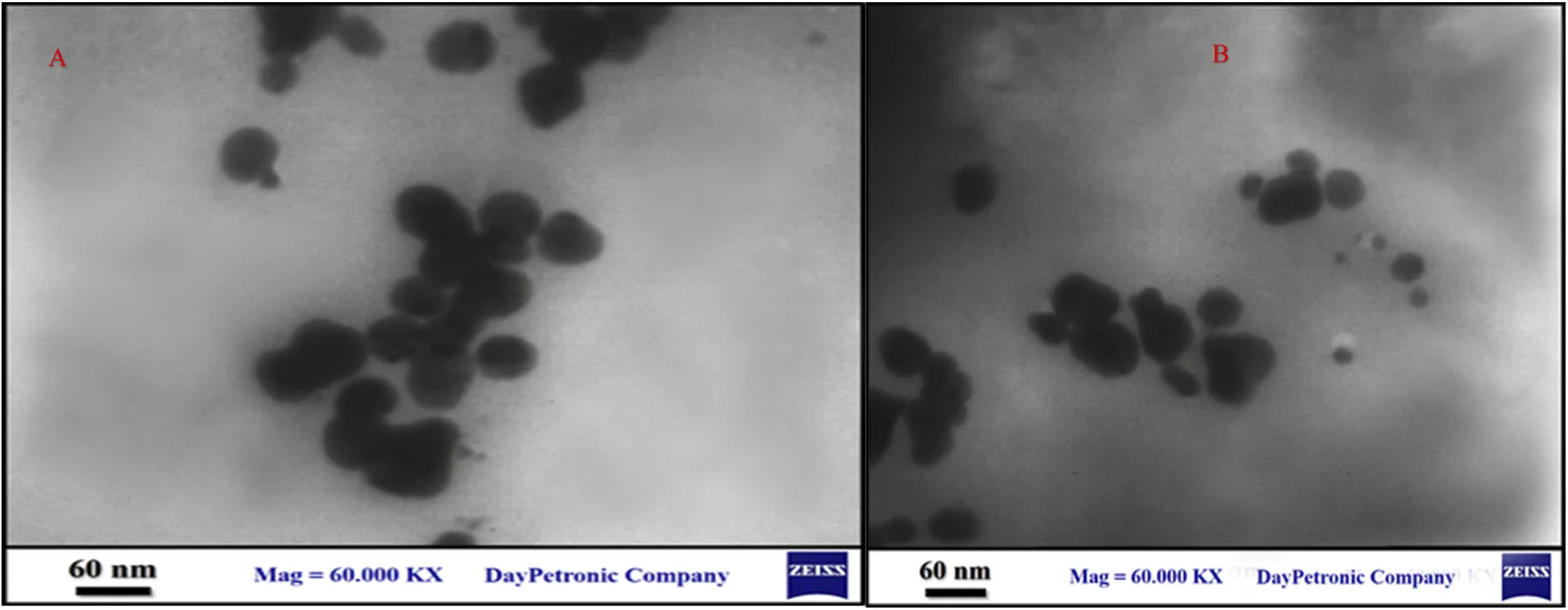
TEM images of AgNPs synthesized using leaf extracts of (A) *Arbutus andrachne* and (B) *Pistacia lentiscus*.

### 3.6 EDX (energy-dispersive X-ray analysis)

Energy-dispersive X-ray (EDX) spectrometers were used to confirm the presence of the elemental silver signal of the silver nanoparticles (Fig. 12). The elemental composition of AgNPs is illustrated in Fig. 12, as determined by EDS, proving the presence of Ag (silver), alongside other organic components within the nanoparticles.^31^ The existence of additional components was attributable to the plant extracts. The quantitative examination *via* EDX reported a silver content of 65.7% by weight in the *Pistacia lentiscus*-synthesized silver nanoparticles. As shown in Fig. 12B, the *Pistacia lentiscus* extract-produced silver nanoparticles include the following weight percentages of elements: silver: 65.7%, carbon: 25.77%, oxygen: 7.18%, aluminum: 0.74%, and silicon: 0.6%. The weight percentages of elements in *Arbutus andrachne*-synthesized AgNPs (Fig. 12A) are as follows: carbon: 54.75%, oxygen: 25.28%, silver: 15.97%, potassium: 1.39%, calcium: 1.31%, magnesium: 1.2%, and zinc: 0.11%. The low signals of Ca, Mg, Zn, Al, and Si were ascribed to the coating material and the bioactive components of the plant extract.^32^

**Fig. 12:**
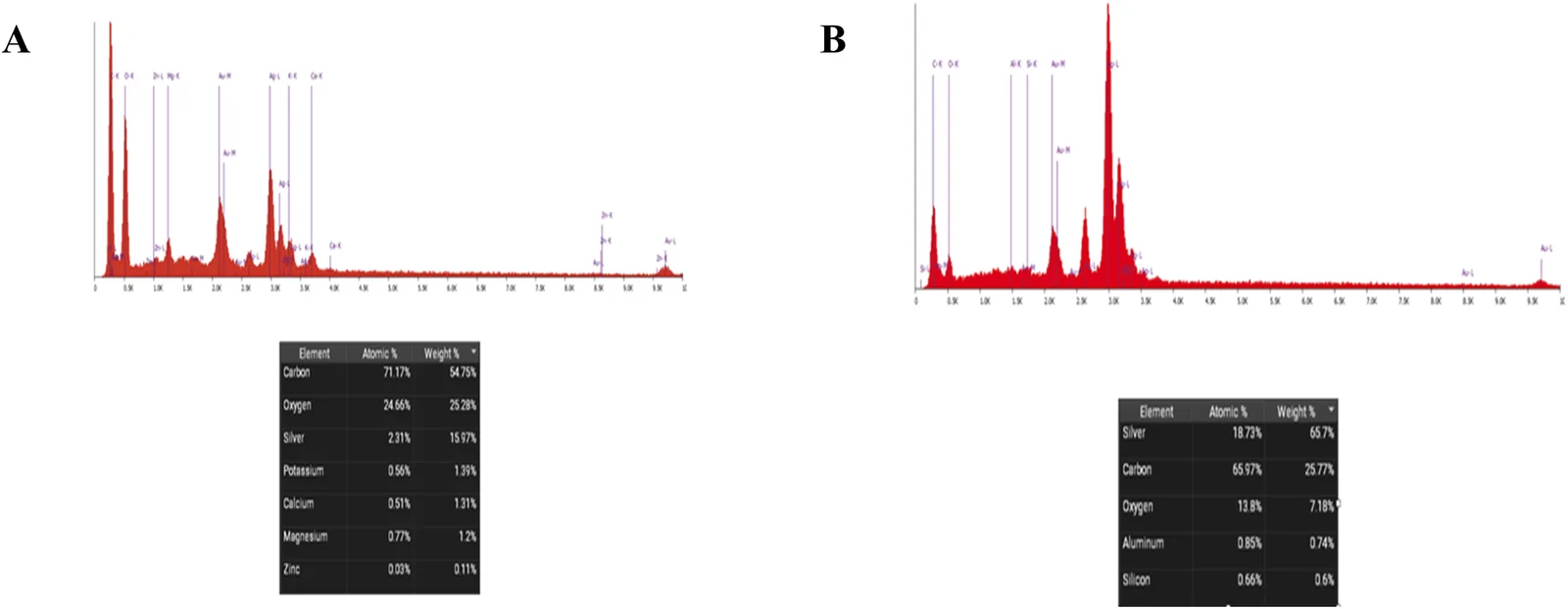
EDX characterization spectra obtained for (A) *Arbutus andrachne* AgNPs and (B) *Pistacia lentiscus* AgNPs.

The EDX study results (Fig. 12) indicated that the *Arbutus andrachne* and *Pistacia lentiscus* plants exhibited variations in silver (Ag) and carbon (C) content, attributable to variances in phytochemical compositions and metal-ion affinity. *Arbutus andrachne* plant extract-synthesized AgNPs exhibited a moderate silver content of 34% and a comparatively elevated carbon content of 25%, likely attributable to its organic matrix, which was rich in carbonaceous compounds, such as flavonoids and polyphenols, promoting silver-nanoparticle synthesis while potentially restricting excessive silver deposition. *Pistacia lentiscus* plant extract-produced AgNPs demonstrated a markedly elevated silver content of 65% and a reduced carbon content of 20%, indicating enhanced metal-chelating capacity and superior nucleation efficiency for silver accumulation. The diminished carbon % in *Pistacia lentiscus* may suggest a low presence of organic stabilizers or variations in the plant's phytochemical composition, resulting in increased silver absorption and deposition. The compositional variations underscored the unique biochemical interactions between metal ions and plant extracts, affecting the efficiency of nanoparticle synthesis and the distribution of elements.^33,34^

### 3.7 XRD (X-ray diffraction)

The crystalline structure of AgNPs was validated by X-ray diffraction (XRD), as shown in Fig. 13. The XRD spectra for AgNPs synthesized using the *Pistacia lentiscus* extract and *Arbutus andrachne* extract, as depicted in Fig. 13A and B, exhibited significant peaks at 2*θ* = 36.13°, 44.32°, 65.04°, and 77.34°, as well as 37.73°, 46.06°, 64.64°, and 77.04°, corresponding to the (111), (200), (220), and (311) Bragg reflections of the face-centered cubic structure of silver, respectively.

**Fig. 13:**
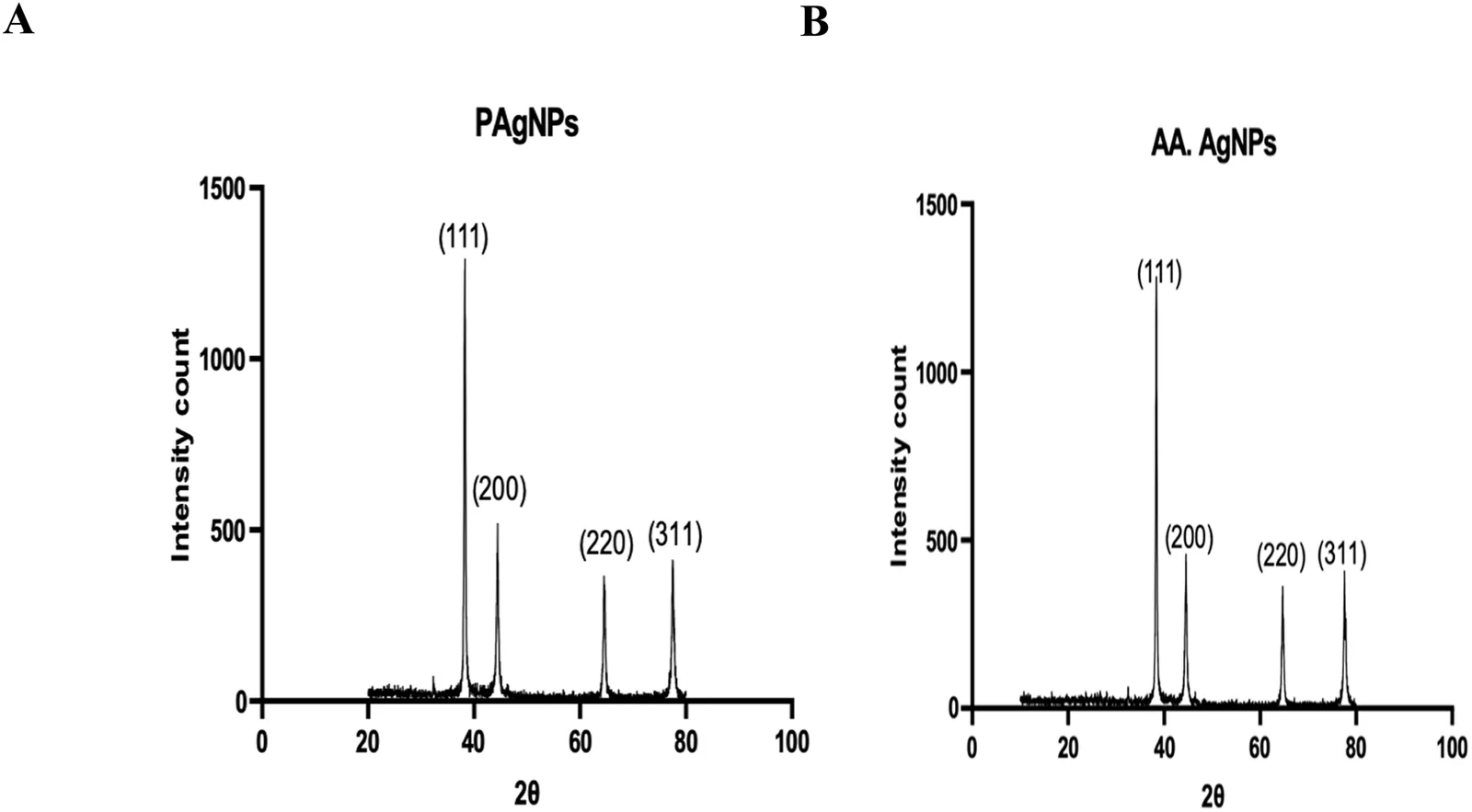
XRD patterns of (A) *Pistacia lentiscus* AgNPs and (B) *Arbutus andrachne* AgNPs.

As reported by Nayak D. *et al.*, the broad diffraction peaks imply a small crystalline size. Additional peaks at 2*θ* values in the AgNPs pattern could be attributed to the remnants of the organic constituents of the plant extract.^35^ The peaks indicated the crystallization of certain plant metabolite moieties on the surface of the AgNPs, corroborating the findings of Shanmuganathan *et al.*.^36^ This constituted valid evidence to verify the role of the plant-extract constituent in the synthesis of AgNPs. This outcome aligned with the results of the XRD analysis conducted by Oves *et al.*^37^

### 3.8 Fourier-transform infrared (FTIR) spectroscopy

FTIR measurements were conducted to identify the functional groups present in the synthesized AgNPs. The binding of biomolecules, which involved capping and stabilizing the NPs, was validated by FTIR spectra in the range of 4000–400 cm^−1^. The FTIR results unveiled distinct bond strains at different peaks (Fig. 14A), which presented a comparison between the *Arbutus andrachne* and *Pistacia lentiscus* plant extract-synthesized silver nanoparticles. FTIR analysis was employed to determine potential bioactive compounds responsible for the reduction and capture of silver ions in reduced *Arbutus andrachne* AgNPs. The FTIR spectra of *Arbutus andrachne* AgNPs exhibited clear peaks at 3336, 2940, 2105, 1626, 1334, 1043, 1025, and 624 cm^−1^. The FTIR spectra of *Arbutus andrachne* AgNPs (Fig. 14A) showed a broad band at 3336 cm^−1^, attributed to O–H and/or N–H stretching vibrations. The band at 2940 cm^−1^ was assigned to the aliphatic C–H stretching of CH_2_ and CH_3_ groups, while the weak band at 2105 cm^−1^ and features around 2200 cm^−1^ were tentatively associated with C≡C stretching. The absorption band at 1626 cm^−1^ was attributed to C=C stretching, whereas bands at 1334, 1043, and 1025 cm^−1^ were associated with oxygen-containing functional groups within the fingerprint region. The band at 624 cm^−1^ was assigned to C–H bending vibrations. Changes in these bands following AgNP formation suggested the involvement of plant-derived functional groups in the reduction of Ag^+^ to Ag^0^ and the subsequent stabilization of the nanoparticles. Therefore, the hydroxyl-containing constituents of *A. andrachne* may contribute to its reducing and antioxidant properties during AgNP synthesis.^38^

**Fig. 14:**
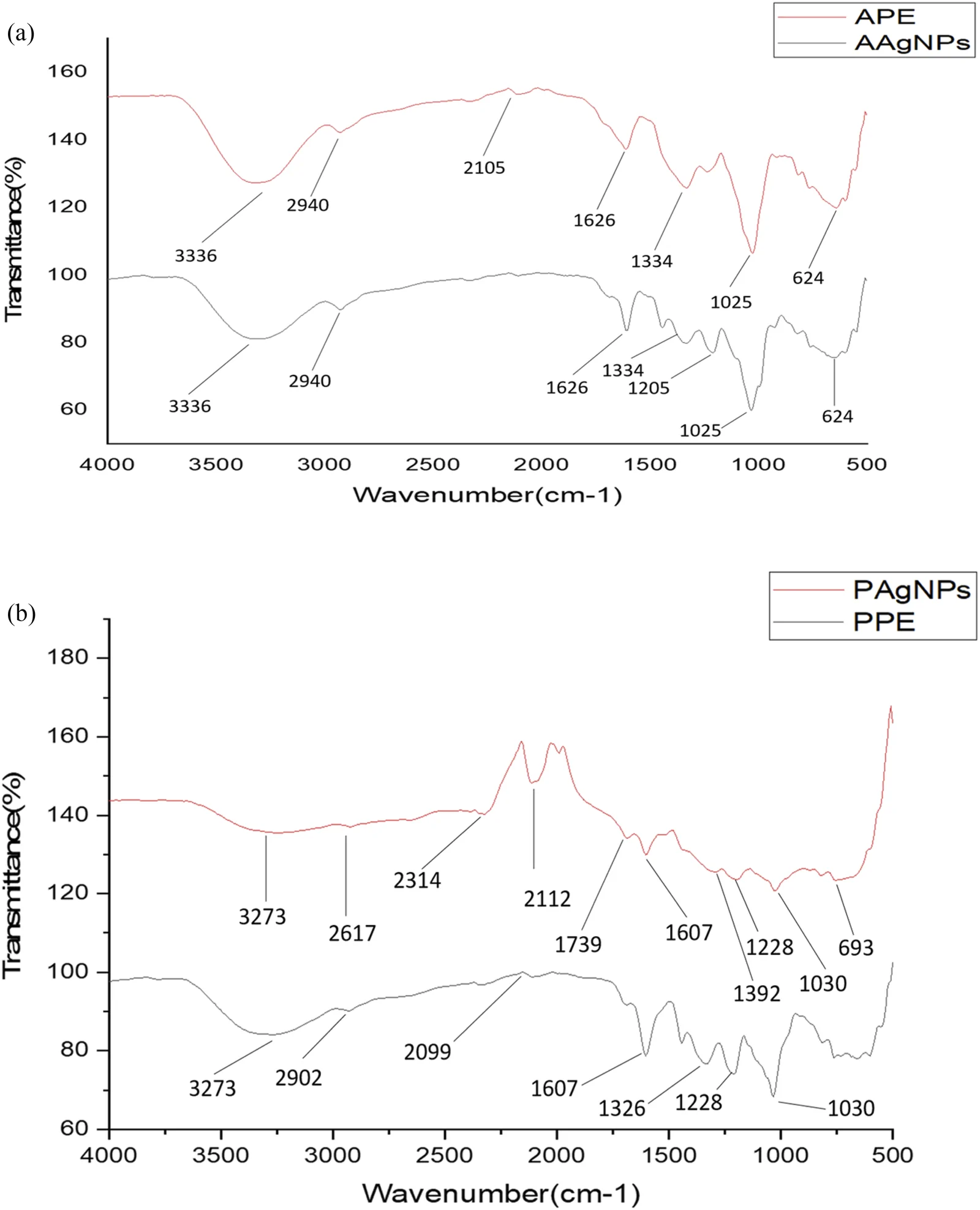
FTIR spectra of the plant extracts and corresponding synthesized silver nanoparticles (AgNPs): (A) *Arbutus andrachne* extract and A.A-AgNPs; and (B) *Pistacia lentiscus* extract and PL-AgNPs.

As shown in Fig. 14B, the band at 2940 cm^−1^ was attributed to aliphatic C–H stretching, while the weak absorption at 2105 cm^−1^ was tentatively assigned to C≡C stretching. The band at 624 cm^−1^ may correspond to the deformation vibrations of α-glucopyranose rings in carbohydrate-containing compounds,^39^ whereas the low-frequency band at 448 cm^−1^ may be associated with skeletal or bending vibrations within the fingerprint region.

Fig. 14B revealed the functional groups in the leaf extract-produced AgNPs, which were identified from several spectral peaks at distinct wavelengths. The minor peaks at 1228 cm^−1^ and 1392 cm^−1^ corresponded to the C–O stretching or C–N stretching vibrations of amine esters in the extract. The band detected at 663.47 cm^−1^ may result from the deformation of α-glucopyranose rings in carbohydrates.^40^

The broad absorption band observed at 3273 cm^−1^ was assigned to overlapping O–H and N–H stretching vibrations, indicating the presence of hydroxyl- and amine-containing constituents derived from the *Pistacia lentiscus* extract and adsorbed water. The N–O stretching vibration of nitro compounds was observed at approximately 1392 cm^−1^, while the N–H stretching vibration of primary amines was detected around 1607 cm^−1^. Comparable research exhibited a distinct diagnostic functional classification (*i.e.*, O–H alcoholic/acid and C–O–C). The relative shift in intensity distribution and peak positions in the IR spectra, as depicted in Fig. 14B, indicated that various functional groups in the plant extract likely contributed to AgNP formation and were crucial for capping and stabilization, thereby inhibiting AgNP aggregation.^41^

### 3.9 Antioxidant activity

#### 3.9.1 DPPH (2, 2-diphenyl-1-picrylhydrazyl)

In order to investigate the antioxidant activities of AgNPs of *Arbutus andrachne* and *Pistacia lentiscus*, the DPPH scavenging assay was conducted. Silver nanoparticles (AgNPs) synthesized from *Pistacia lentiscus* and *Arbutus andrachne* extracts demonstrated substantial free radical scavenging activity against DPPH, as illustrated in Fig. 15. AgNPs, particularly those synthesized from *Pistacia lentiscus*, showed significant scavenging capacity and an antioxidant efficacy comparable to that of ascorbic acid, a recognized antioxidant. This DPPH assay validated the substantial antioxidant activity of AgNPs synthesized from the aqueous extract of *Pistacia lentiscus* and the methanol extract of *Pistacia lentiscus*. The elevated activity of *Pistacia lentiscus* AgNPs could be ascribed to the existence of several functional groups, as indicated by FTIR analysis, which facilitated the bioreduction of Ag ions into AgNPs. This indicated the potential utility of AgNPs as an alternative antioxidant in the therapy of free radical-induced diseases. Numerous studies have demonstrated the significant antioxidant activity of AgNPs synthesized from plant extracts (Tables 3 and 4).^42–44^

**Fig. 15:**
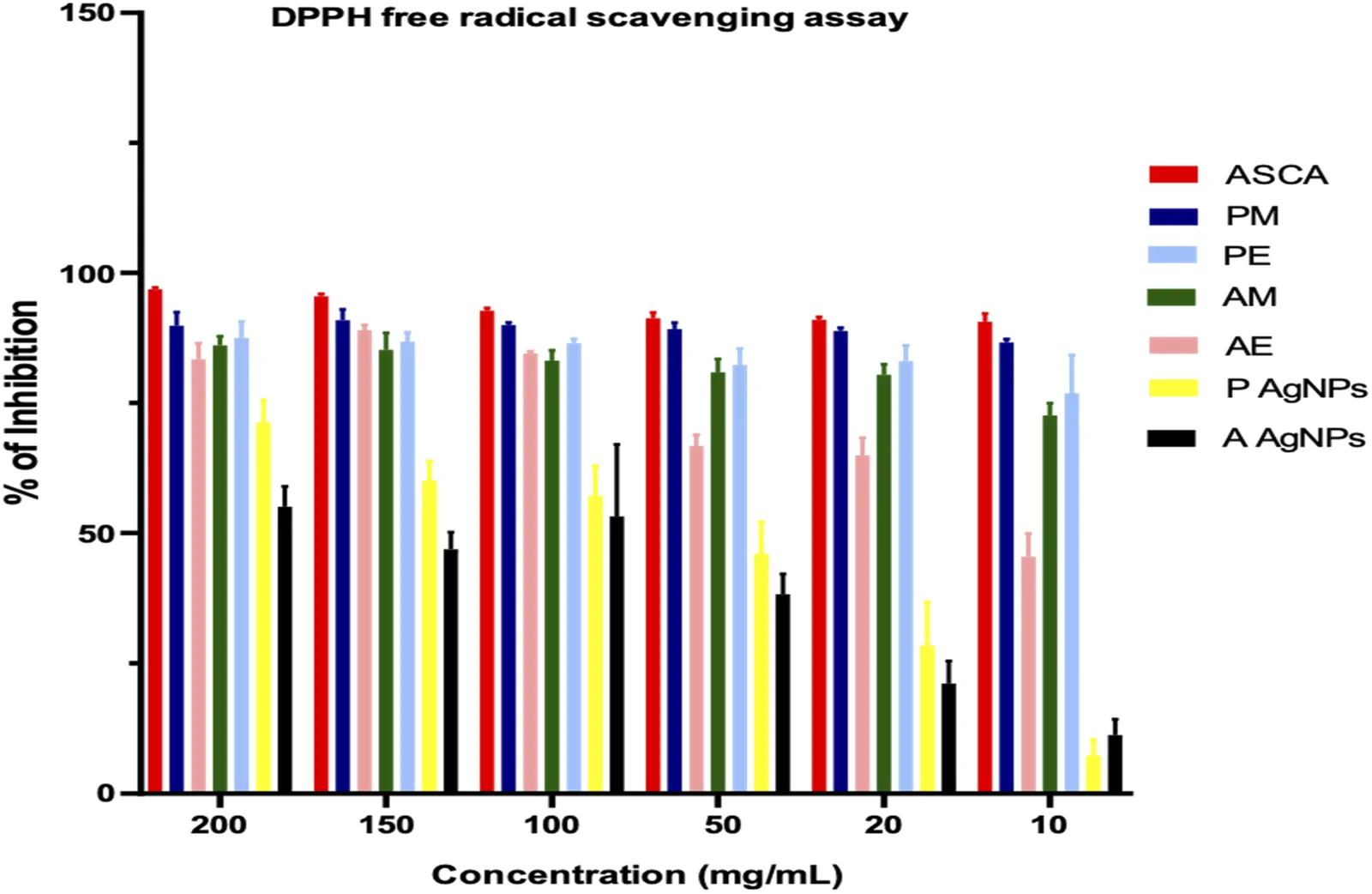
Inhibition (%) of concentration values of ascorbic acid, PM, PE, AM, AE, A.A AgNPs, and P.L AgNPs.

**Table 3:** Concentration values of ascorbic acid, PM, PE, AM, AE, A.A AgNPs and P.L AgNPs using UV-vis spectrometry

Concentration (µg L^−1^)	Ascorbic acid	PM	PE	AM	AE	A.A AgNPs	P.L AgNPs
10	0.119	0.171	0.302	0.273	0.212	0.758	0.873
20	0.106	0.151	0.689	0.333	0.293	1.260	0.979
50	0.128	0.149	0.355	0.288	0.323	0.829	0.951
100	0.102	0.146	0.690	0.363	0.345	1.294	1.690
150	0.097	0.168	0.713	0.437	0.306	1.657	1.980
200	0.082	0.152	1.117	0.553	0.336	2.129	1.995

**Table 4:** Inhibition (%) of concentration values of ascorbic acid, PM, PE, AM, AE, A.A AgNPs, and P.L AgNPs

Concentration (µg L^−1^)	Ascorbic acid	PM	PE	AM	AE	A.A AgNPs	P.L AgNPs
10	94.66	92.33	86.46	87.76	90.49	66.01	60.85
20	95.25	93.22	69.10	85.07	86.86	43.50	56.10
50	94.26	93.31	84.08	87.08	85.52	62.83	57.35
100	95.43	93.45	69.06	83.72	84.53	41.97	24.22
150	95.65	92.47	68.03	80.40	86.28	25.70	11.21
200	96.32	93.18	49.91	75.20	84.93	4.53	10.54

*Pistacia lentiscus* comprises many substances, including alkaloids, phenols, and proteins, which can donate hydrogen from their hydroxyl group (–OH) to free radicals, resulting in the formation of stable phenoxyl radicals. The scavenging properties of AgNPs extracted from *Arbutus andrachne* and *Pistacia lentiscus* were determined by the DPPH scavenging method because the antioxidant capacity of compounds was directly proportional to their reducing power. DPPH was a stable compound that received electrons or hydrogen from AgNPs. The findings from the DPPH assay demonstrated significant free radical suppression by both AgNPs and the plant extract (Fig. 15). Across the tested concentration range (10–200 µg L^−1^), neither *Arbutus andrachne* AgNPs nor *Pistacia lentiscus* AgNPs exhibited a concentration-dependent increase in inhibition. Instead, inhibition generally declined with increasing concentration, despite a temporary increase at 50 µg L^−1^. Overall, inhibition decreased from 66.01% to 4.53% for *A. andrachne* AgNPs and from 60.85% to 10.54% for *P. lentiscus* AgNPs. Comparable findings indicated that the enhanced DPPH scavenging activity was attributed to the silver nanoparticles.^45^ The findings validated that the plant extract, AgNPs, and ascorbic acid exhibited antioxidant actions. It was also observed that the plant extract exhibited superior antioxidant activity relative to ascorbic acid.

### 3.10 Antimicrobial

This study investigated the antibacterial efficacy of manufactured AgNPs against pathogenic (harmful) bacteria utilizing the disc diffusion method on two bacterial types: *Enterococcus faecalis* (Gram-positive) and *Escherichia coli* (Gram-negative). Fig. 16 illustrates petri dish images, which contain agar, bacterial colonies, and discs with varying amounts of AgNPs following 24 h of incubation. All examined bacteria showed resistance to plant extracts that were diluted equally with water (Table 5). Moreover, silver nitrate (100 µg mL^−1^, 200 µg mL^−1^, and 300 µg mL^−1^) exhibited considerable antibacterial activity. The results for precursor concentrations of 100 µg mL^−1^ and 200 µg mL^−1^, together with the final concentration of 300 µg mL^−1^ for both *Arbutus andrachne* and *Pistacia lentiscus* AgNPs, are displayed in Tables 5 and 6, respectively. The values derived using azithromycin as a positive control were considered more effective than those of the synthesized AgNPs and methanol and ethanol plant extracts.

**Fig. 16:**
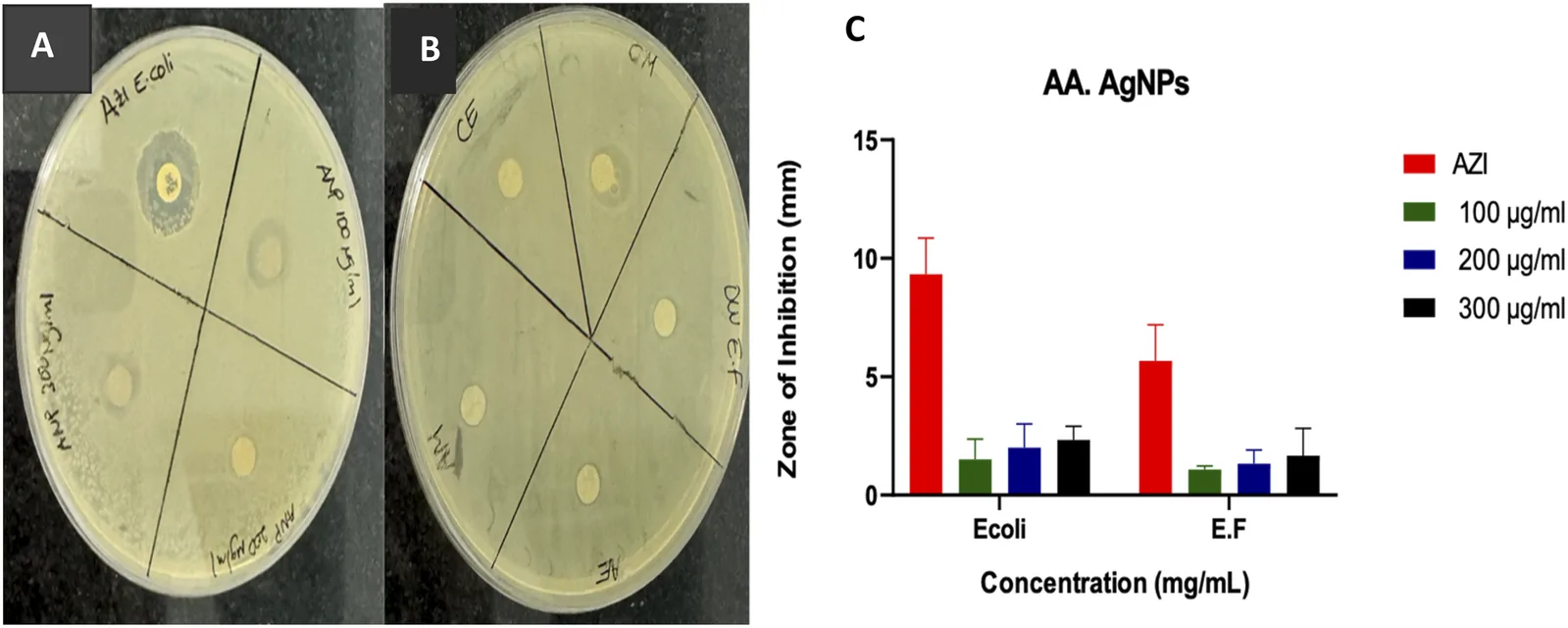
Antimicrobial analysis of (A) *E. coli* treated with *Arbutus andrachne* AgNPs, (B) *Enterococcus faecalis* treated with plant extract and distill water (DW), and (C) antibacterial inhibition of A.A AgNPs (AZI, 100 µg mL^−1^, 200 µg mL^−1^, and 300 µg mL^−1^).

**Table 5:** Results for *Arbutus andrachne* AgNPs (AZI, 100 µg mL^−1^, 200 µg mL^−1^, and 300 µg mL^−1^) cultured with *Escherichia coli* (*E. coli*) and *Enterococcus faecalis* (*E. faecalis*)^a^

Bacterial strain	AZI (mm)	A.A AgNPs (mm) 100 µg mL^−1^	A.A AgNPs (mm) 200 µg mL^−1^	A.A AgNPs (mm) 300 µg mL^−1^	*P*-value
*E. coli*	9.33 ± 1.53	1.50 ± 0.87	2.00 ± 1.00	2.33 ± 0.58	0.001
*E. faecalis*	5.67 ± 1.53	1.08 ± 0.14	1.33 ± 1.53	1.67 ± 1.57	0.002

a Values are presented as mean ± SD (*n* = 3). Inhibition zones are expressed in mm. Statistical significance was determined using one-way ANOVA, with *p* < 0.05 considered statistically significant.

**Table 6:** Results for *Pistacia lentiscus* AgNPs (AZI, 100 µg mL^−1^, 200 µg mL^−1^, and 300 µg mL^−1^) cultured with *Escherichia coli* (*E. coli*) and *Enterococcus faecalis* (*E. faecalis*)^a^

Bacterial strain	AZI (mm)	P.L AgNPs (mm) 100 µg mL^−1^	P.L AgNPs (mm) 200 µg mL^−1^	P.L AgNPs (mm) 300 µg mL^−1^	*P*-value
*E. coli*	11.33 ± 1.15	1.18 ± 0.28	1.67 ± 0.58	3.67 ± 1.53	0.0001
*E. faecalis*	7.50 ± 1.32	0.73 ± 0.64	1.33 ± 0.58	2.33 ± 1.15	0.001

a Values are presented as mean ± SD (*n* = 3). Inhibition zones are expressed in mm. Statistical significance was determined using one-way ANOVA, with *p* < 0.05 considered statistically significant.

The antibacterial study demonstrated that *Arbutus andrachne* AgNPs and *Pistacia lentiscus* AgNPs prepared with various concentrations of plant extracts (100 µg mL^−1^, 200 µg mL^−1^, and 300 µg mL^−1^) exhibited antibacterial activity. The results indicated that AgNPs synthesized from plant extracts exhibited varying antibacterial activity against different bacterial strains. Overall, all examined samples exhibited antibacterial activity comparable to that of standard azithromycin, with the exception of 100 µg mL^−1^*Arbutus andrachne* AgNPs. *Pistacia lentiscus* and *Arbutus andrachne* methanol plant extracts exhibited antibacterial activity comparable to that of the positive control, azithromycin.

*Pistacia lentiscus* AgNPs were the most efficacious of the AgNPs synthesized using plant extract. This pertained to the discovery that the antibacterial efficacy was contingent upon the dimensions and morphology of AgNPs.^46^ The antibacterial efficacy of nanoparticles prepared from 300 µg mL^−1^ AgNPs was higher than that of those prepared from 100 µg mL^−1^ AgNPs, as demonstrated in Tables 5 and 6.

Sankar and colleagues also reported the correlation between antibacterial activity and AgNP concentration for various bacteria.^46,47^ As shown in Fig. 17, *Enterococcus faecalis* exhibited the highest resistance to all forms of produced AgNPs and methanol and ethanol plant extracts. The observation may be attributed to the bacterial cellular morphology, as it was a Gram-positive bacterium. Other researchers have similarly shown low activity against Gram-positive bacteria.^48–50^

**Fig. 17:**
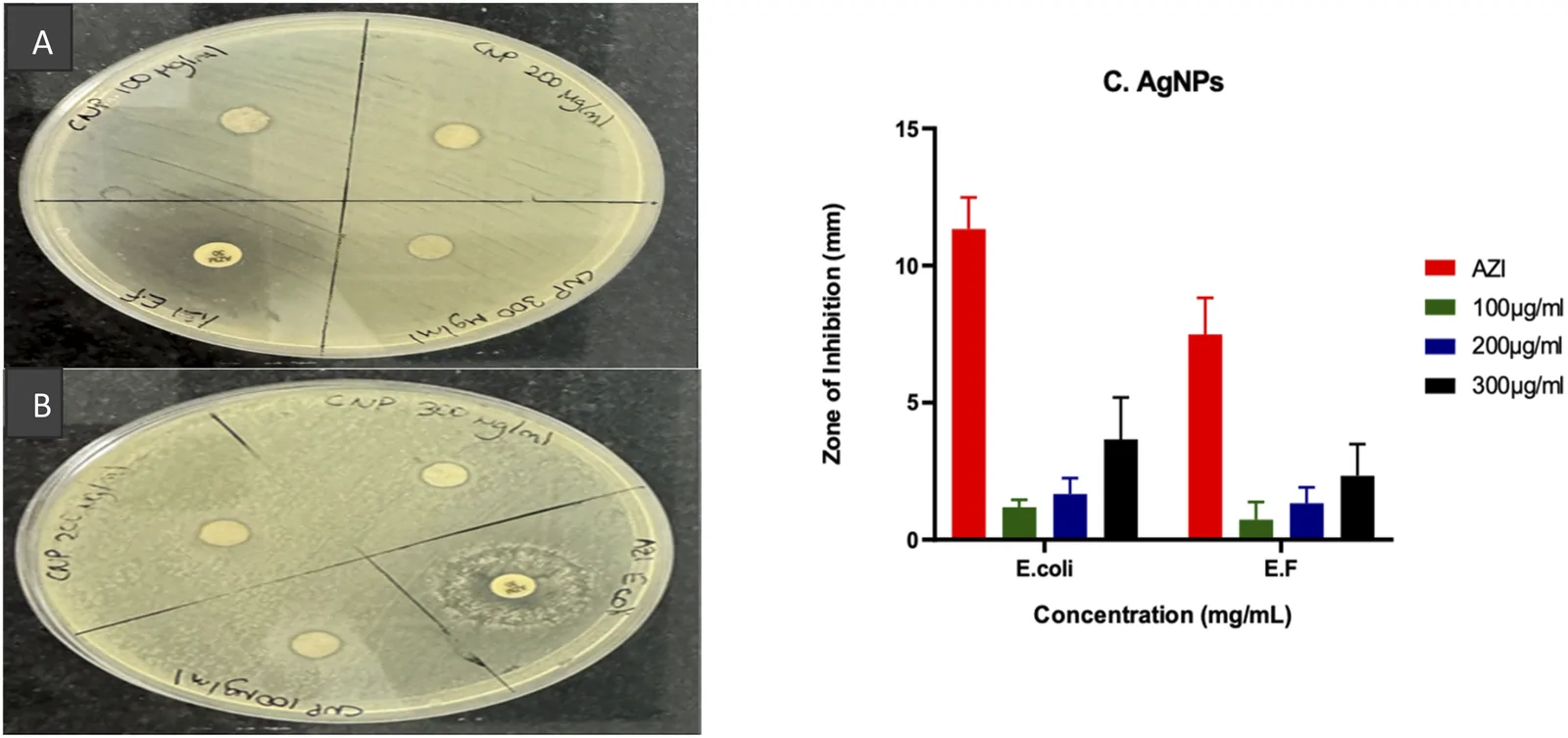
Antimicrobial analysis of (A) *E. coli* treated with *Pistacia lentiscus* AgNPs (B) *Enterococcus faecalis* treated with *Pistacia lentiscus* AgNPs and (C) antibacterial inhibition of *Pistacia lentiscus* AgNPs (AZI, 100 µg mL^−1^, 200 µg mL^−1^, and 300 µg mL^−1^).

As illustrated in Fig. 18, the increased vulnerability of Gram-negative bacteria, in contrast to Gram-positive bacteria, is attributed to their more accessible penetration. In the case of *Escherichia coli*, as shown in Fig. 17, synthesized AgNPs at 300 µg mL^−1^ from plant extracts derived from *Pistacia lentiscus* and *Arbutus andrachne* exhibited more activity than for *Enterococcus faecalis*. Therefore, it could be inferred that biosynthetically produced AgNPs from different plant extracts acted as antibacterial agents, with the efficiency depending on the concentration. Similarly, it was found that nanoparticles synthesized from the aqueous extract of black mustard seeds (*B. nigra*) had efficacy against the examined bacteria, *Propionibacterium acnes*, *Klebsiella pneumoniae*, and *Pseudomonas aeruginosa*,^51^ which was similar to the results of this research study. A prior report indicated that AgNPs synthesized from lavender leaves exhibited antibacterial action against *Escherichia coli*, *Salmonella typhi*, *Proteus mirabilis*, *Staphylococcus aureus*, *Bacillus cereus*, *Pseudomonas aeruginosa*, and *Klebsiella oxytoca*.^52^

**Fig. 18:**
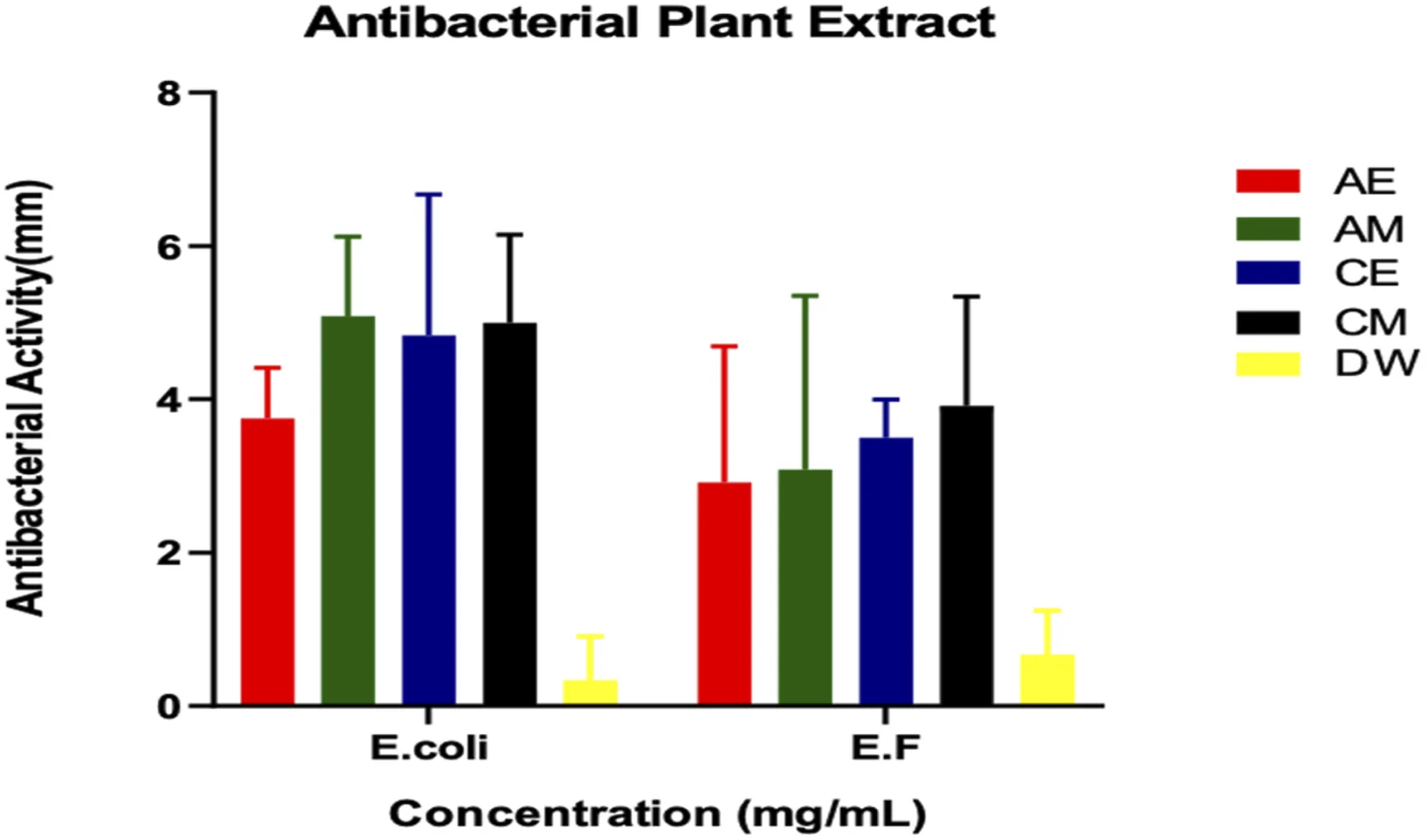
Results for the plant AE, AM, PE, PM, and DW extracts cultured with *Escherichia coli* (*E. coli*) and *Enterococcus faecalis* (*E. faecalis*).

As shown in Fig. 18, the antibacterial activity of AgNPs is contingent upon a few factors: AgNP concentration, plant species, and bacterial species. The antibacterial activity of AgNPs made from plant extracts was significantly above that of the pure extract, which exhibited low to no antibacterial activity (Table 7). The high activity was possibly due to the discharge of the silver ions from the AgNPs, which caused the destruction of cell walls and secondary metabolites attached to the surface of AgNPs.^53–55^ The high bacterial activity observed could also be because of the activity of the silver ions in the forms of AgCl formed or AgNO_3_ left unreacted. Some biologically active compounds in the organic matrix could increase the antibacterial activity of the AgNPs.

**Table 7:** Results for plant AE, AM, PE, PM, and DW extracts cultured with *Escherichia coli* (*E. coli*) and *Enterococcus faecalis* (*E. faecalis*)^a^

Bacterial strain	AE (mm)	AM (mm)	PE (mm)	PM (mm)	DW (mm)	*P*-value
*E. coli*	3.75 ± 0.66	5.08 ± 1.04	4.83 ± 1.84	5.00 ± 1.15	0.33 ± 0.58	0.002
*E. faecalis*	2.92 ± 1.77	3.08 ± 2.27	3.50 ± 0.50	3.92 ± 1.42	0.67 ± 0.58	0.142

a Values are presented as mean ± SD (*n* = 3). Inhibition zones are expressed in mm. Statistical significance was determined using one-way ANOVA, with *p* < 0.05 considered statistically significant.

### 3.11 Cytotoxicity assessment of the plant-synthesized silver nanoparticles

The cytotoxic activities of AgNPs synthesized by two different medicinal plants (*P. lentiscus* and *A. andrachne*) were analyzed through a cell viability assay, using concentrations ranging between 0 and 500 µg mL^−1^ (Fig. 19). The results indicated that both samples had significant cytotoxic properties, since cell viability significantly decreased with increasing concentrations of AgNPs. The cell viability of AgNPs obtained from *P. lentiscus* at 50 µg mL^−1^ (around 92%) was higher than that of *A. andrachne* (74%) AgNPs, thereby indicating a comparatively higher cytotoxic effect. At a concentration of 250 µg mL^−1^, the cell viability dropped to around 82% for *P. lentiscus* and 49% for *A. andrachne* AgNPs, where the difference was statistically significant (**p < 0.001). At the maximum concentration of 500 µg mL^−1^, the significant cytotoxic effects of both samples reduced the cell viability to around 45% and 36%, respectively.

**Fig. 19:**
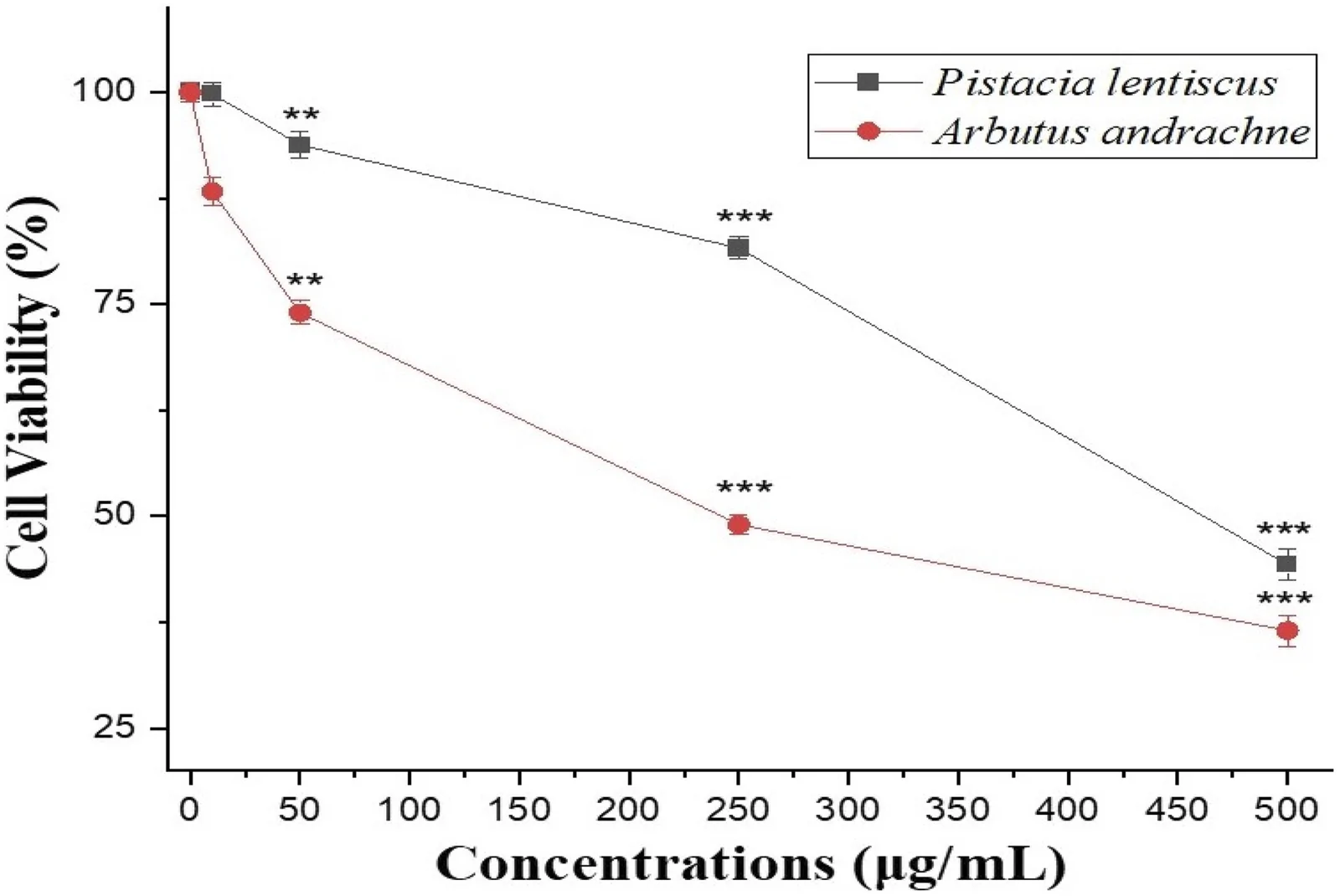
Cytotoxicity of *Pistacia lentiscus*- and *Arbutus andrachne*-synthesized AgNPs.

The dose-dependent cytotoxic effect was similar to earlier findings about green-synthesized silver nanoparticles, which exhibited various anticancer activities through oxidative stress, mitochondria-related damage, and apoptosis stimulation.^56^ The small particle size, along with a large surface area, increased the interaction between the AgNPs and cell components, thereby facilitating the production of high amounts of reactive oxygen species.

It was worth noting that AgNPs extracted from *Arbutus andrachne* proved more efficacious as cytotoxins compared to the nanoparticles obtained from *Pistacia lentiscus*. Such increased activity could be associated with dissimilarities in plant metabolites responsible for nanoparticle formation through reduction and capping processes.^57^ In addition to playing a role in the synthesis of nanoparticles, the bioactive substances produced by plants could act synergistically with nanoparticles and increase their activity. Since there was a noticeable decrease in cell viability with the increase in concentrations from 250 to 500 µg mL^−1^, it may be assumed that AgNPs affected the essential mechanisms of living cells, such as DNA synthesis, protein synthesis, and other processes that affect cell membranes. Similar observations were made previously from research conducted on green-synthesized AgNPs, which showed excellent anticancer effects against various cancer lines in a dose-dependent manner.^58^

The cytotoxic activity of AgNPs may involve several interconnected cellular mechanisms. Following cellular uptake, AgNPs can release Ag^+^ ions and promote the generation of reactive oxygen species (ROS), resulting in oxidative stress and subsequent cellular damage. Elevated ROS levels may impair mitochondrial function, alter mitochondrial membrane potential, and initiate apoptosis-related pathways. AgNPs may also interact with cellular proteins and DNA, potentially disrupting essential processes involved in cell survival and proliferation. In addition, phytochemicals associated with the nanoparticle surface may influence these biological effects through their interactions with the AgNPs. However, these mechanisms are proposed based on reported effects of AgNPs, as ROS generation, mitochondrial dysfunction, and apoptosis were not directly investigated in the present study.

Overall, AgNPs synthesized using both *P. lentiscus* and *A. andrachne* exhibited cytotoxic activity against MDA-MB-231 cells, with *A. andrachne*-derived AgNPs showing greater activity than *P. lentiscus*-derived AgNPs. This difference may be associated with variations in the phytochemical compositions of the plant extracts and their interaction with the nanoparticle surface. Although the findings indicate the anticancer potential of these plant-mediated AgNPs, further investigation using additional cancer cell lines, different exposure periods, ROS measurements, mitochondrial membrane-potential analysis, and apoptosis assays is required to clarify the underlying mechanisms and establish their therapeutic relevance.

## 4. Conclusion

DLS analysis showed *Z*-average hydrodynamic diameters of 90.87 nm and 78.26 nm for *Arbutus andrachne*- and *Pistacia lentiscus*-derived AgNPs, respectively, whereas TEM indicated particle sizes of approximately 60 nm. This variation was expected because DLS measured the hydrodynamic size of nanoparticles in suspension, as well as the surrounding solvation layer and surface-associated biomolecules, while TEM measured the physical dimensions of the dried particles. Consequently, the particle sizes obtained by DLS were generally larger than those observed by TEM. Nonetheless, techniques such as gas chromatography mass spectrometry and Fourier transform infrared spectroscopy could be utilized to detect or identify various biomolecules in the extracts of *Arbutus andrachne* and *Pistacia lentiscus*. The biosynthesized AgNPs were analyzed by UV-visible spectroscopy, X-ray diffraction, scanning electron microscopy, and transmission electron microscopy. SEM and TEM images proved the surface morphology and the size distribution of the synthesized AgNPs. Microbial studies confirmed the antibacterial effect of the plant-loaded AgNPs against *E. coli* and *E. faecalis* bacteria. The biological performance of the synthesized AgNPs may depend on both particle size and the phytochemical constituents of the plant extracts. Small nanoparticles provide a high surface-area-to-volume ratio, which may increase their interactions with biological targets and promote Ag^+^ ion release, thereby enhancing antimicrobial and cytotoxic effects. Furthermore, phytochemicals derived from *Pistacia lentiscus* and *Arbutus andrachne* may serve as reducing and stabilizing agents during AgNP formation and may remain associated with the nanoparticle surface, potentially contributing to the observed biological activities. For further studies, the plant concentrations can be increased for better antimicrobial efficiency. Both *Pistacia lentiscus* and *Arbutus andrachne* extracts exhibited promising anticancer activities, with *A. andrachne* showing superior efficacy. These results highlighted their potential as natural sources of anticancer agents. However, further investigations, including mechanistic studies, isolation of active compounds, and *in vivo* validation, are necessary to fully elucidate their therapeutic potential. The differences observed in the antibacterial, antioxidant, and cytotoxic activities of the two AgNP formulations may arise from the combined effects of particle size, surface characteristics, and differences in the phytochemical constituents of the respective plant extracts.

Future projections for the environmentally friendly synthesis of nanoparticles indicate a significant increase in applications; however, there is a pressing need to address the long-term impacts on animals and humans, as well as the environmental accumulation of these particles. Biogenic nanoparticles can be utilized as nanoweapons for targeting phytopathogens and in diverse water disinfection methods for environmental remediation. In medication-delivery systems, these nanoparticles may represent a pivotal advancement for the biomedical sector.

## Author contributions

Doga Kavaz: writing – review & editing, writing – original draft, validation, supervision, project administration, methodology, investigation, funding acquisition, formal analysis, data curation, conceptualization. Temitayo Margaret Omoyeni: writing – review & editing, writing – original draft, validation, resources, methodology, investigation, funding acquisition, formal analysis, data curation, conceptualization. Buse Taskiranlar: writing – original draft, validation, resources, methodology, investigation, funding acquisition, formal analysis, data curation, conceptualization. Huzaifa Umar: writing – original draft, validation, resources, methodology, investigation, funding acquisition, formal analysis, data curation, conceptualization.

## Conflicts of interest

No potential conflicts of interest were reported by the author(s).

## Data Availability

All relevant data are included in the paper.
